# Single‐Cell Transcriptome Profiling Reveals Conserved IFNγ‐IL8 Signaling‐Induced Antibacterial Neutrophil States during Bacterial Infection

**DOI:** 10.1002/advs.202504840

**Published:** 2025-07-01

**Authors:** Xue Zhai, Minghao Zhang, Kang Li, Wei Li, Xiaolong Li, Wa Gao, Zhaosheng Sun, Dan Huang, Songqian Huang, Mingli Liu, Zhichao Wu, Jun Zou, Liangbiao Chen, Jialong Yang, Peng Hu

**Affiliations:** ^1^ Key Laboratory of Exploration and Utilization of Aquatic Genetic Resources Ministry of Education Shanghai Ocean University Shanghai 201306 China; ^2^ International Research Center for Marine Biosciences Ministry of Science and Technology Shanghai Ocean University Shanghai 201306 China; ^3^ Center for Aquacultural Breeding Research Shanghai Ocean University Shanghai 201306 China; ^4^ State Key Laboratory of Estuarine and Coastal Research School of Life Sciences East China Normal University Shanghai 200241 China; ^5^ The State Key Laboratory of Grassland Agro‐ecosystems College of Pastoral Agriculture Science and Technology Lanzhou University Lanzhou Gansu 730020 China

**Keywords:** IFNγ‐IL8 signaling, immune response, neutrophils, publicly accessible dataset, single‐cell transcriptomic profiles

## Abstract

*Streptococcus agalactiae* is a significant pathogen in both humans and animals, yet the immune cell subtype dynamics during infection remain poorly defined. Leveraging the high susceptibility and tractability of Nile tilapia (*Oreochromis niloticus*), 113,356 single immune cells are profiled from head kidney and spleen across multiple infection time points (0, 1, 5, 10, 75 days post‐infection and 3 days post‐reinfection). This single‐cell transcriptomic and flow cytometry analyses revealed distinct activation and transition patterns among neutrophils, macrophages, T cells, and B cells. Neutrophils exhibited early transcriptional remodeling enriched in inflammatory and interferon gamma (IFNγ) signaling pathways. Cross‐species integration identified a conserved IFNγ‐driven transition toward il8⁺ neutrophils. Furthermore, recombinant interleukin‐8 (IL8) enhanced antibacterial responses in tilapia and human neutrophils, while inhibition of STAT1 reduced *IL8* expression. IL8 stimulation increased phagocytosis and  reactive oxygen species (ROS） production, supporting its role in neutrophil‐mediated bacterial clearance. Together, this findings establish IFNγ‐IL8 as a conserved mechanism in vertebrate immunity and a potential target for antibacterial therapies.

## Graphical abstract

**Summary**This study constructs a single‐cell immune atlas of Nile tilapia across multiple time points during *Streptococcus agalactiae* infection, revealing dynamic immune cell responses and subtype transitions. It identifies a conserved IFNr‐STAT1‐IL8 axis driving il8^+^ neutrophils activation and antibacterial function, supported by cross‐species validation and functional assays, with implications for vertebrate immunity.

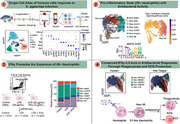

## 1 Introduction

*Streptococcus agalactiae* (Group B Streptococcus, *S. agalactiae*) is a significant pathogen impacting human health‐especially neonates, pregnant women, and immunocompromised individuals‐and also causes disease in economically important aquaculture species like Nile tilapia (*Oreochromis niloticus*).^[^ ^1^, ^2^, ^3^, ^4^, ^5^ ^]^ Nile tilapia's high susceptibility and practical advantages make it an ideal model to investigate host immune responses to *S. agalactiae* infection, aligning with the One Health framework that addresses shared disease mechanisms across species.^[^ ^6^, ^7^, ^8^ ^]^ Despite its global impact, the mechanistic understanding of host immunity to *S. agalactiae* remains limited, with few effective treatments beyond antibiotics.

Immune defense involves diverse cell types, but conventional approaches lack the resolution to dissect cellular heterogeneity and state transitions during infection.^[^ ^9^, ^10^, ^11^, ^12^, ^13^ ^]^ Single‐cell RNA sequencing (scRNA‐seq) offers unprecedented insight into immune complexity and has advanced host‐pathogen studies in mammals.^[^ ^13^, ^14^, ^15^ ^]^ Although scRNA‐seq has been applied to teleost fish, including tilapia, the characterization of specific immune cell subsets and their functional dynamics during bacterial infection is still incomplete.^[^ ^16^, ^17^, ^18^, ^19^, ^20^, ^21^ ^]^ Among immune cells, neutrophils are essential innate effectors conserved across vertebrates and exhibit significant functional heterogeneity. In mammals, interferon gamma (IFNγ) activates pro‐inflammatory neutrophil subsets with enhanced antimicrobial activity,^[^ ^22^, ^23^, ^24^, ^25^, ^26^, ^27^, ^28^, ^29^ ^]^ but whether similar neutrophil subtype specialization occurs in teleosts as well as which molecular mechanisms drive this enhanced antimicrobial function during *S. agalactiae* infection remain unclear.

To address this gap, we performed scRNA‐seq profiling of over 113,000 immune cells from Nile tilapia at six time points following *S. agalactiae* infection, revealing dynamic shifts in immune populations and neutrophil subtype transitions linked to enhanced antibacterial function. Cross‐species integration with human data and functional assays further uncovered a conserved IFNγ‐driven emergence of an il8⁺ neutrophil subtype characterized by increased phagocytosis and reactive oxygen species (ROS) production. Our findings establish IFNγ‐IL8 signaling as a key regulator of antibacterial immunity. The publicly accessible dataset (https://hulabshou.shinyapps.io/tilapia/) provides a valuable resource for comparative immunology and neutrophil‐focused host defense research.

## 2 Results

### 2.1 scRNA‐seq Atlas Reveals Dynamic Immune Cell Response to *S. agalactiae* Infection in Nile Tilapia

**Figure 1:**
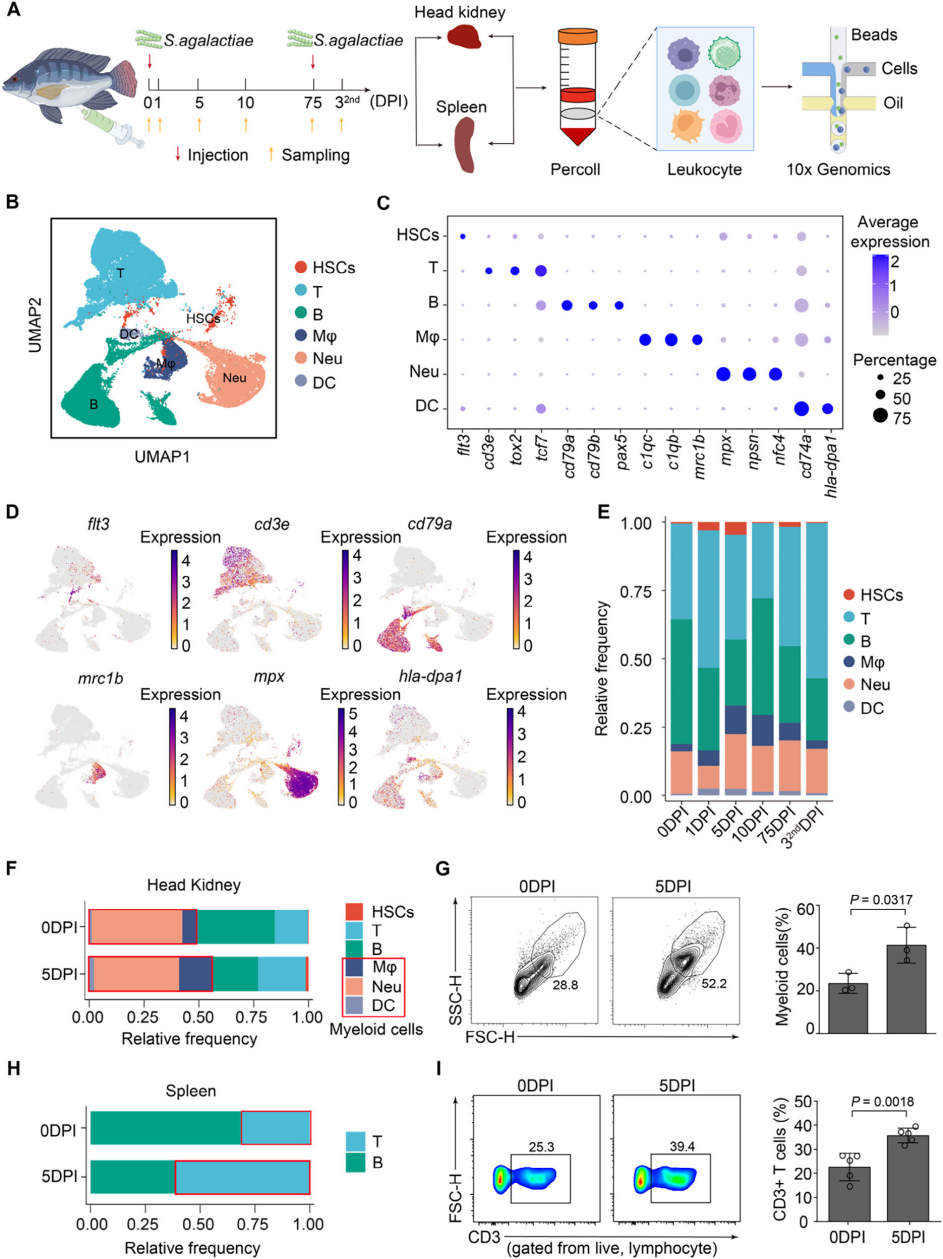
Profiling of the 81,004 single cells isolated from six‐time points of head kidney and spleen after *S. agalactiae* infection in the Nile tilapia. （A) Schematic of the overall study design. DPI: days post‐infection. (B) UMAP plot of the integrated all‐time points datasets, with cells colored according to the major cell types. HSCs: hematopoietic stem cells, T: T cells, B: B cells, Mφ: macrophages, Neu: neutrophils, DC: dendritic cells. (C) Dot plots showing the expression of the classical marker genes used to define each major cell type, colored by the average expression of each gene in each cluster scaled across all clusters, dot size indicates the percentage of cells in each cluster expressing the corresponding gene. (D) UMAP visualization of classical marker genes, with cells colored according to the normalized expression level of the corresponding genes. (E) Bar plot showing the cell proportion of each major cell type across six‐time points. Color represents the cell type. (F) Bar plots showing the composition of myeloid cells of the head kidney at 0 DPI and 5 DPI. Color represents the cell type. DPI: days post‐infection. Red box indicated the myeloid cells. (G) Representative flow cytometry plots (left) and bar plots (right) showing the frequency of myeloid cells in head kidney. DPI: days post‐infection. Data in bar plots are mean ± SEM, each dot represents an individual animal (*n* = 3). Significance was determined by Student's *t*‐test. (H) Bar plots showing the composition of T cells of spleen at 0 DPI and 5 DPI. Color represents the cell type. DPI: days post‐infection. (I) Representative flow cytometry plots (left) and bar plots (right) showing the frequency of CD3^+^ T cells. DPI: days post‐infection. Data in bar plots are mean ± SEM, each dot represents an individual animal (*n* = 5). Significance was determined by Student's *t*‐test.

To analyze the immune cell composition and dynamics in Nile tilapia, we generated scRNA‐seq data from the spleen and head kidney of control and bacteria‐infected fish. Samples were collected at six‐time points: 0, 1, 5, 10, 75 days post‐infection (DPI), and 3 days post‐reinfection (**Figure** **1**A). After quality control, filtering for cells with 200–4,000 genes and less than 15% mitochondrial gene content, we retained 81,004 high‐quality cells: 15,512 from 0 DPI, 7,277 from 1 DPI, 14,783 from 5 DPI, 16,456 from 10 DPI, 13,151 from 75 DPI, and 13,825 from 3 days post‐reinfection (Figure S1A, Supporting Information). Data validation through integrating with another scRNA‐seq dataset of tilapia head kidney tissue^[^ ^16^ ^]^ confirmed consistency and detected even more genes (Figure S1A–F, Supporting Information).

We identified 9 cell clusters using unsupervised clustering (Figure S1A, Supporting Information), including hematopoietic stem cells (HSCs), T cells (T), B cells (B), macrophages (Mφ), neutrophils (Neu), dendritic cells (DCs), endothelial cells (Endo), platelet, and erythrocyte (Ery). Focusing on 6 major clusters, we dissected immune cell heterogeneity: hematopoietic stem cells (HSCs) (*flt3*), T cells (*cd3e*, *tox2*, and *tcf7*), B cells (*pax5*, *cd79a*, and *cd79b*), macrophages (Mφ) (*c1qc*, *c1qb*, and *mrc1b*), neutrophils (Neu) (*mpx*, *npsn*, and *ncf4*), and dendritic cells (DCs) (*cd74a* and *hla‐dpa1*) (Figure 1,B,C). Marker gene expression patterns in uniform manifold approximation and projection (UMAP) confirmed specific cell type identification (Figure 1D).

While the overall cell distribution remained stable across time points, the proportions of immune cell types shifted dynamically in response to infection (Figure 1E; Figure S1G, Supporting Information). T and B cells dominated the immune landscape, but macrophages, neutrophils, and T cells markedly expanded at 5 DPI‐a trend validated by flow cytometry in both the head kidney and spleen (Figure 1,E–I). This likely reflects increased pathogen clearance and cell turnover, with neutrophils and macrophages removing debris and T cells supporting adaptive immunity.^[^ ^30^ ^]^ Although exact proportions differed between scRNA‐seq and flow cytometry‐possibly due to limitations in FSC/SSC‐based gating (Figure S1H)‐both methods revealed consistent trends. Notably, a sharp rise in T cells at 3 days post‐reinfection (Figure 1E) highlights the role of memory T cells in mounting a rapid secondary immune response.

### 2.2 Neutrophil Dynamics and Functional Specialization During *S. agalactiae* Infection

**Figure 2:**
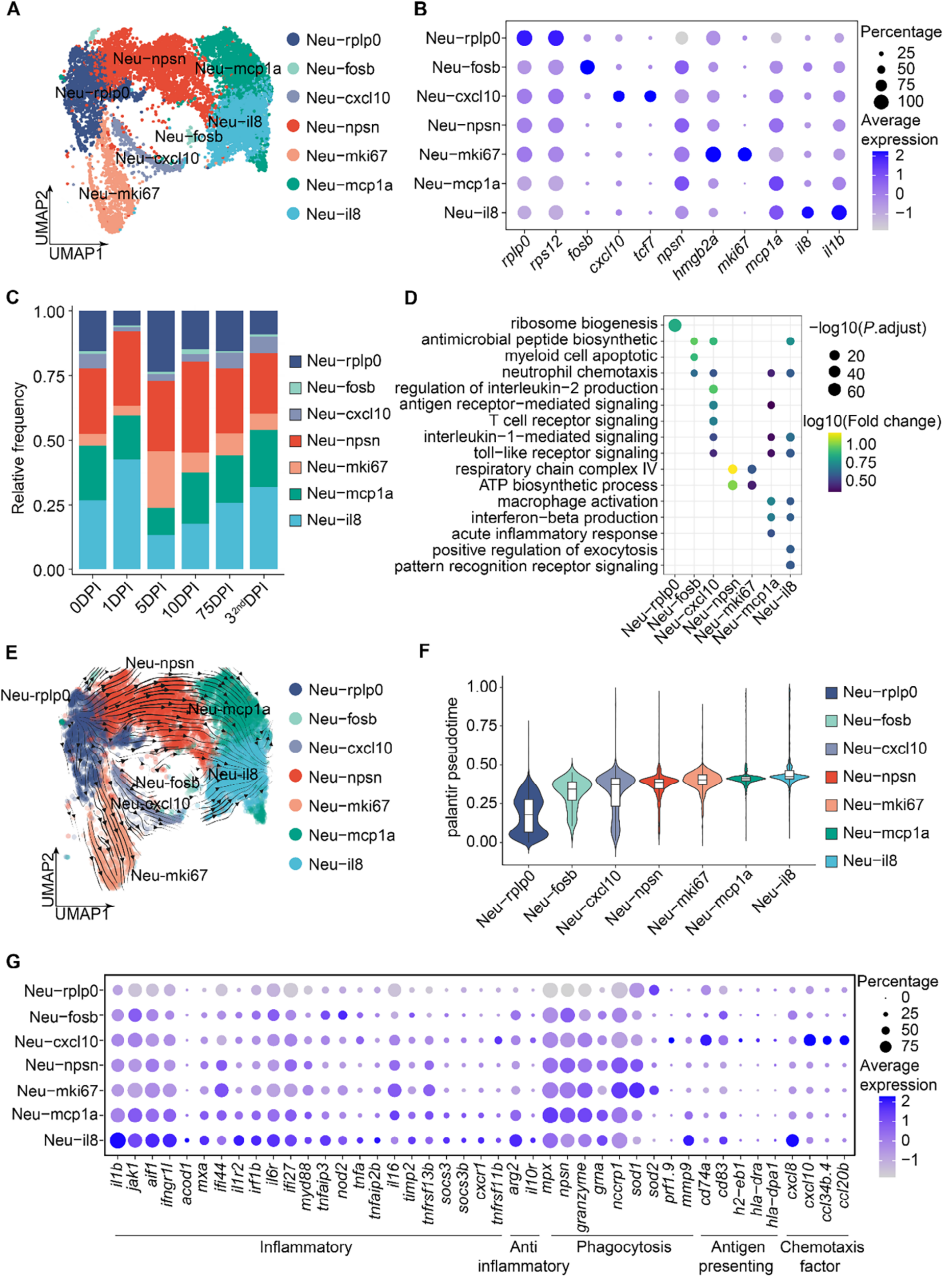
Characterization of neutrophil subpopulations and dynamics. (A) UMAP plots of neutrophils, with cells colored according to neutrophil subtypes. (B) Dot plot showing the top marker genes in each neutrophil subtype. Colors represent the average expression of each gene in each cluster, scaled across all clusters. Dot size indicates the percentage of cells in each cluster expressing the corresponding gene. (C) Bar plot showing the proportions of the seven neutrophil clusters across six‐time points. DPI: days post‐infection. Color represents the cell type. (D) Dot plot illustrating GO enrichment of marker genes for each subtype. The significance of enrichment is determined by a hypergeometric test. Dot size is scaled by −log10 (false discovery rate (FDR)‐adjusted *P*‐value) and colored by log10 (fold change). Fold change is calculated as the fraction of DEGs found in a pathway divided by the fraction of all expressed genes within the pathway. (E) UMAP visualization of neutrophil subtype transitions by RNA velocity analyses. The streamlines indicate the integration paths that connect local projections from the observed state to the extrapolated future state. Arrows indicate the directionality of subtype transition computed by CellRank. Cells are color‐coded by subtypes. (F) Violin plot illustrating the pseudotime distribution of each neutrophil subtype inferred using the Palantir algorithm in CellRank, with Neu‐rplp0 as the root node. (G) Expression of different gene sets (inflammatory, anti‐inflammatory, phagocytosis, antigen‐presenting, chemotaxis factors) in each neutrophil subtype. Colors represent the average expression of each gene in each cluster, scaled across all clusters. Dot size indicates the percentage of cells in each cluster expressing the corresponding gene.

Extensive studies in mammals have shown that neutrophils are highly heterogeneous, with diverse functions and origins.^[^ ^24^, ^25^ ^]^ In Nile tilapia, we observed a sharp decline in neutrophil proportions at 1 day post‐infection (DPI), dropping from 14.86% to 7.33%, followed by a gradual recovery (Figure 1E). This early depletion suggests dynamic changes in neutrophil states in response to infection, prompting a deeper exploration of their transcriptional diversity. By leveraging scRNA‐seq to dissect this heterogeneity and reveal the continuous state transition dynamics of neutrophils in teleost, we identified seven distinct subtypes based on their gene expression profiles: Neu‐rplp0 (*rplp0* and *rps12*), Neu‐fosb (*fosb*), Neu‐cxcl10 (*cxcl10* and *tcf7*), Neu‐npsn (*npsn*), Neu‐mki67 (*hmgb2a* and *mki67*), Neu‐mcp1a (*mcp1a*), and Neu‐il8 (*il8* and *il1b*) (**Figure** **2**A,B).

At 1 DPI, the Neu‐il8 subtype expanded, showing enrichment in the Gene Ontology (GO) terms related to exocytosis regulation, interferon‐beta production, and acute inflammatory response, indicating their role in inflammation, consistent with previous findings (Figure 2C,D). Neu‐mki67, characterized by cell cycle and proliferation genes (*hmgb2a* and *mki67*), increased significantly at 5 DPI (Figure 2C,D).

Although neutrophils are traditionally considered mature and terminally differentiated,^[^ ^25^ ^]^ their maturation states in teleosts remain uncertain. Using CellRank pseudotime analysis,^[^ ^31^ ^]^ we uncovered a continuous differentiation pathway from Neu‐rplp0 to Neu‐il8, with Neu‐il8 representing the most terminal state (Figure 2E,F; Figure S2A–C, Supporting Information). This trajectory was further validated using other algorithms, including Monocle,^[^ ^32^ ^]^ CytoTRACE,^[^ ^33^ ^]^ and Palantir^[^ ^34^ ^]^ (Figure S2A, Supporting Information). Pseudotime analysis revealed distinct functional modules, with inflammatory genes such as *il8*, *il1b*, *tnip2*, *atf3*, and *marcksl1b* showing high expression at the terminal state (Figure S2D, Supporting Information). Gene expression analysis revealed that inflammatory genes were highly enriched in the Neu‐il8 subset, indicating its specialized role in the antibacterial inflammatory response (Figure 2G). Given IL8's established function in immune cell recruitment, we used CellPhoneDB^[^ ^35^ ^]^ to assess neutrophil interactions with other immune cells. This analysis showed strong crosstalk with macrophages (Figure S2E, Supporting Information), primarily mediated by nr3c1‐il8 and il8‐cxcr2 signaling (Figure S2F, Supporting Information), likely coordinating effector cell recruitment during infection.

### 2.3 Temporal Dynamics and Functional Heterogeneity of Macrophages During *S. agalactiae* Infection

To characterize macrophage responses during infection, we analyzed temporal gene expression changes. The highest number of DEGs occurred at 1 and 5 DPI, enriched in cytokine and phagocytosis pathways, while later stages (75 DPI and post‐reinfection) showed upregulation of antigen presentation genes (e.g., *hla‐dpb1*, *cd74*), indicating a shift toward adaptive immunity (Figure S3A,B, Supporting Information).

**Figure 3:**
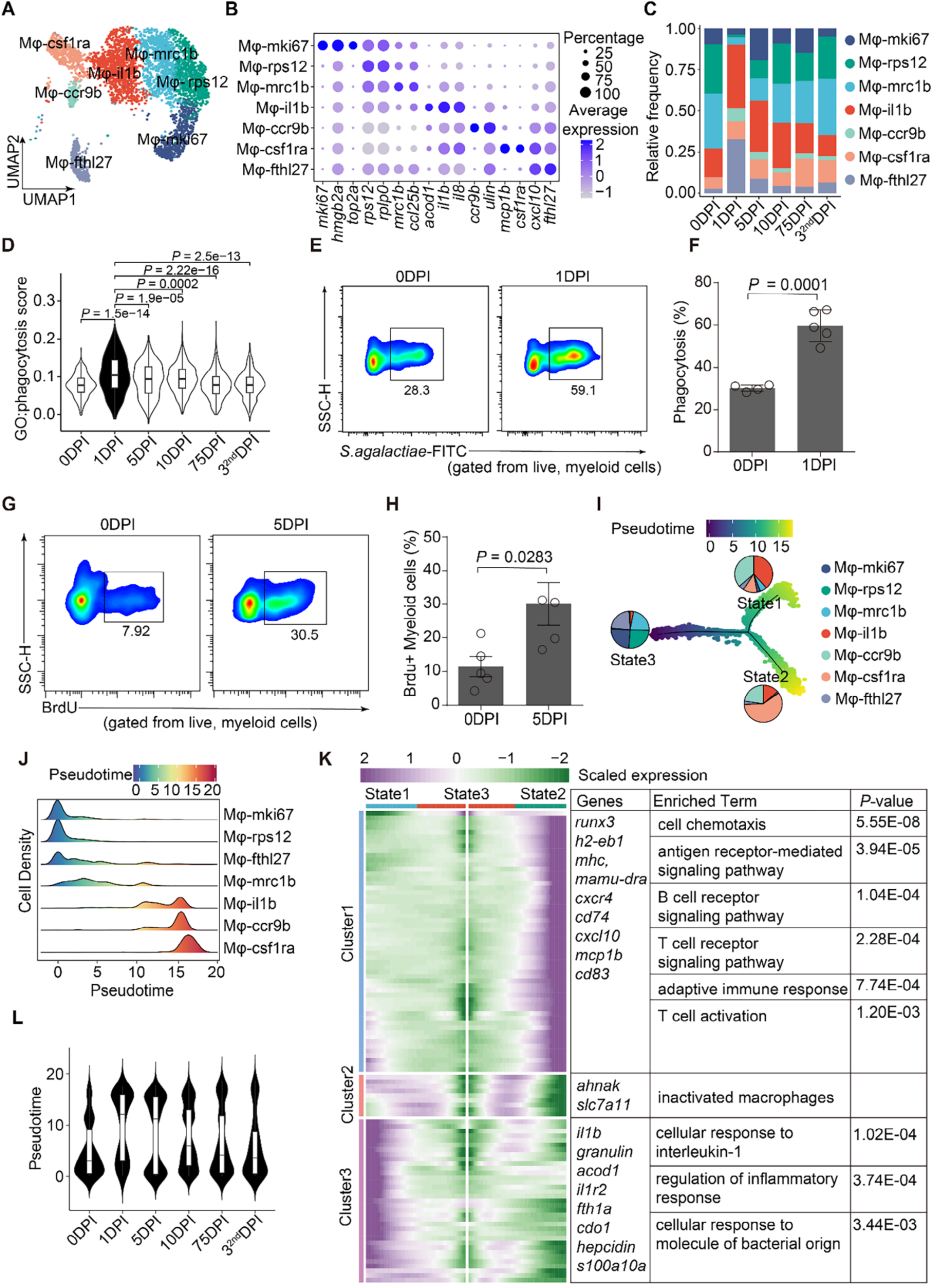
Characterization of macrophage subpopulations and dynamics. (A) UMAP plots of macrophage subtypes, with cells colored according to macrophage subtype. (B) Dot plot showing the top marker genes in each macrophage subtype. Colors represent the average expression of each gene in each cluster, scaled across all clusters. Dot size indicates the percentage of cells in each cluster expressing the corresponding gene. (C) Bar plot showing the proportions of the seven macrophage subtypes across six‐time points. DPI: days post‐infection. Color represents the cell type. (D) Violin plot of phagocytosis signaling pathway scores (GO:0 006911) for macrophages at six‐time points. In the box plot within each violin plot, middle lines indicate median values, boxes range from the 25th to 75th percentiles, and upper/lower whiskers extend from the hinge to the largest/smallest value no further than 1.5 times the interquartile range (IQR) from the hinge. Significance was determined by a two‐sided Wilcoxon rank‐sum test. DPI: days post‐infection. (E) Representative flow cytometry plots of phagocytosing myeloid cells at 0 DPI and 1 DPI. DPI: days post‐infection. (F) Bar plot showing the frequency of phagocytosing myeloid cells. DPI: days post‐infection. Data are presented as mean ± SEM; each dot represents an individual animal (*n* = 4–5). Significance was determined by Student's *t*‐test. (G) Representative flow cytometry plots of BrdU^+^ macrophages at 0 DPI and 5 DPI. DPI: days post‐infection. Positive and negative BrdU^+^ macrophages were gated from myeloid cells. (H) Bar plot showing the frequency of BrdU^+^ macrophages. DPI: days post‐infection. Data are mean ± SEM; each dot represents an individual animal (*n* = 4–5). Significance was determined by Student's *t*‐test. (I) Trajectory analysis of bacterial‐exposed and unexposed macrophages using Monocle. Pseudotime is color‐scaled. Pie plot showing the proportions of macrophage subtypes in each state, with cell types color‐coded. (J) Cell density pseudotemporal patterns of macrophage subtype dynamics. The pseudotime of each cell was estimated using the Monocle algorithm. Colors indicate transcriptional states. (K) Heatmap of DEGs, ordered based on their common kinetics through pseudotime using Monocle. Clusters were identified by hierarchical clustering. DEGs, enriched GO terms, and their *P*‐values associated with each model are shown on the right. Significance was determined by a hypergeometric test. (L) Violin plot of Monocle pseudotime of macrophages across six‐time points. DPI: days post‐infection.

Seven macrophage subtypes were identified based on marker genes (**Figure** **3**A,B), each showing distinct temporal dynamics (Figure 3C). Early‐stage macrophages (Mφ‐il1b, Mφ‐ccr9b) were linked to inflammation and chemotaxis, with ccr9b^+^ macrophages appearing rapidly at 1 DPI. Mφ‐mki67 peaked at 5 DPI, associated with proliferation, while Mφ‐csf1ra emerged later and was enriched for immune signaling and T cell activation (Figure 3C; FigureS3C, Supporting Information).

Functional assays confirmed increased phagocytic activity at 1 DPI (Figure 3D–F), and flow cytometry validated cell cycle activity in Mφ‐mki67 (Figure 3G,H). Monocle trajectory analysis revealed three macrophage states: inflammatory (Mφ‐il1b, Mφ‐ccr9b), adaptive (Mφ‐csf1ra), and resting (others) (Figure 3I–K). Temporal mapping showed early infection triggered rapid macrophage activation and differentiation by 1–5 DPI (Figure 3L).

### 2.4 T Cell Heterogeneity, Dynamics, and Functional States During *S. agalactiae* Infection

**Figure 4:**
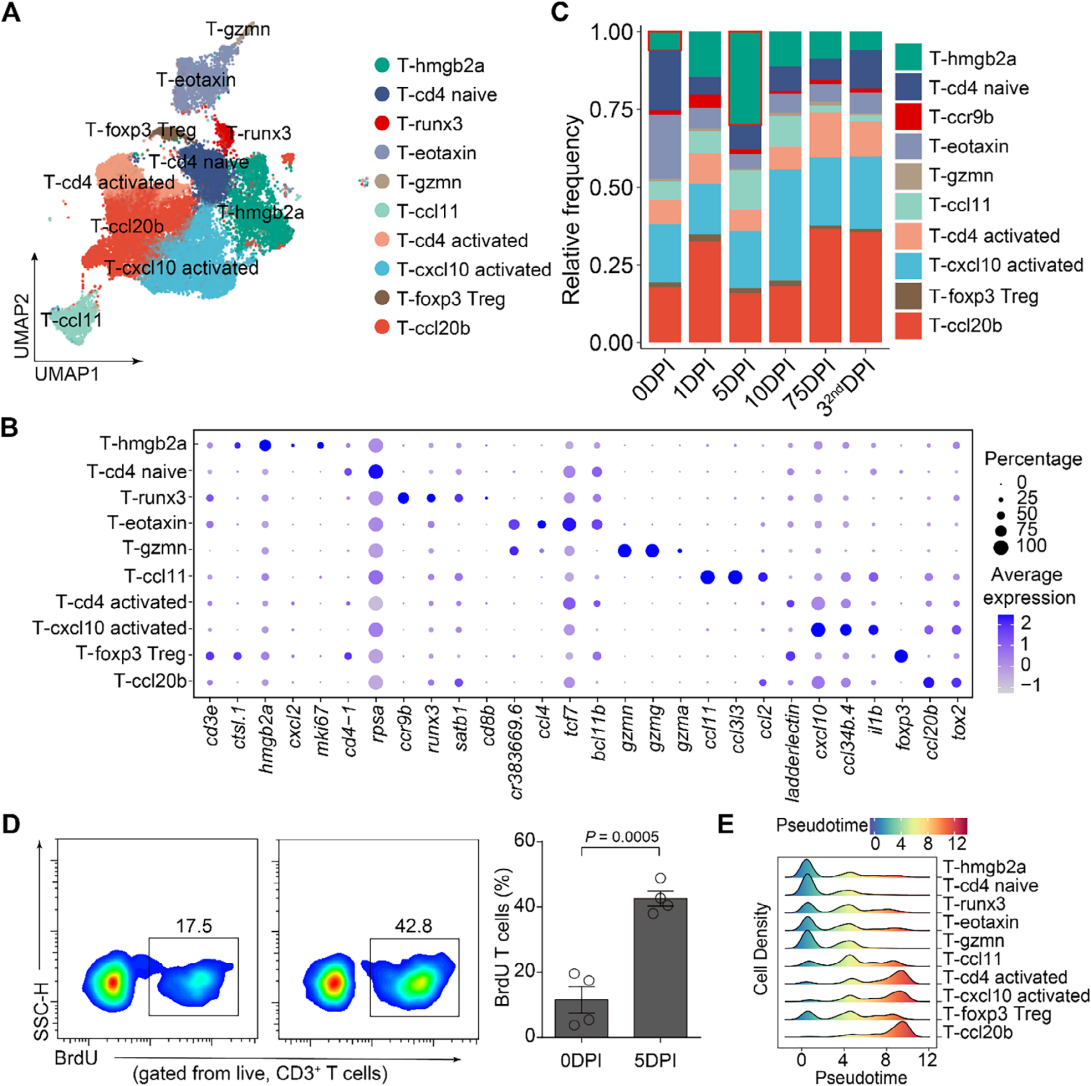
Characterization of T cell subpopulations and dynamics. (A) UMAP plots of T cell clusters, with cells colored according to the T cell subtype. (B) Dot plot showing the expression pattern of classical marker genes of T cell subtypes. Colors represent the average expression of each gene in each cluster, scaled across all clusters. Dot size indicates the percentage of cells in each cluster expressing the corresponding gene. (C) Bar plot showing the proportions of the T cell subtypes across six‐time points. DPI: days post‐infection. Color represents the cell type. (D) Representative flow cytometry plots (left) and bar plots (right) of BrdU^+^ T cells at 0 DPI and 5 DPI. DPI: days post‐infection. Data are mean ± SEM, each dot represents an individual animal (*n* = 4). Significance was determined by Student's *t*‐test. (E) Density plot showing the distribution of the T cell subtypes along the pseudotime. The pseudotime of each cell was estimated by the Monocle algorithm. Colors indicate transcriptional states.

We identified 10 distinct T cell clusters representing diverse functional states and lineages (**Figure** **4**A). These included proliferating cells (T‐hmgb2a), early activation cells (T‐runx3), three CD4^+^ subsets (T‐cd4 naive, T‐cd4 activated, T‐foxp3 Treg), two activation‐associated subsets (T‐ccl20b, T‐cxcl10 activated), and three additional subsets (T‐eotaxin, T‐ccl11, T‐gzmn) characterized by distinct marker gene expression (Figure 4A–C). Temporal distribution analysis revealed dynamic shifts in T cell composition during infection. Proliferating T‐hmgb2a cells peaked at 5 DPI, confirmed by BrdU staining, while T‐runx3 peaked at 1 DPI. Notably, T‐cd4 activated and T‐ccl20b subsets expanded significantly at 75 DPI and post‐reinfection, respectively (Figure 4C,D), indicating context‐specific T cell responses.

Pseudotime trajectory analysis revealed a progression from naive‐like to effector T cells (Figure 4E; Figure S4A, Supporting Information). Early expression clusters included cell cycle genes (*hmgb2a*, *mki67*), followed by mid‐to‐late clusters enriched for interferon‐related genes (*irf9*, *stat5a*, *stat2*) and activation markers, highlighting sequential proliferation and activation (Figure S4B, Supporting Information). These results illustrate the temporal molecular programs and functional diversification of T cells during bacterial infection.

### 2.5 B Cell Dynamics and Function During *S. agalactiae* Infection

**Figure 5:**
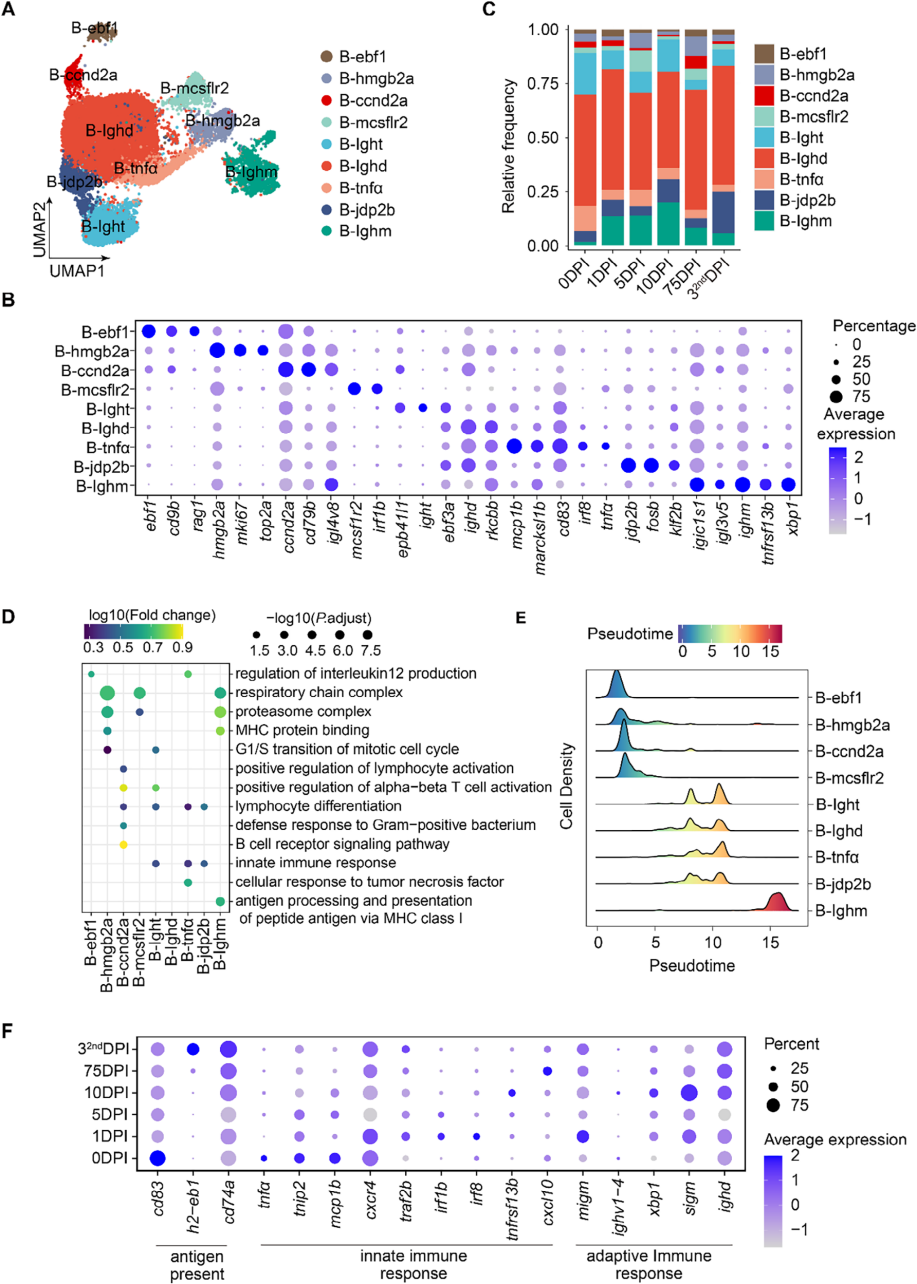
Characterization of B cell subpopulations and dynamics. (A) UMAP plots of B cell clusters, with cells colored according to B cell subtypes. (B) Dot plot showing the expression pattern of top marker genes for B cell subtypes. Colors represent the average expression of each gene in each cluster, scaled across all clusters. Dot size indicates the percentage of cells in each cluster expressing the corresponding gene. (C) Bar plot showing the proportions of B cell subtypes across six‐time points. DPI: days post‐infection. Color represents the cell type. (D) Dot plot showing GO enrichment of marker genes for each subtype. The significance of enrichment is determined by a hypergeometric test. Dot size is scaled by −log10 (false discovery rate (FDR)‐adjusted *P*‐value) and colored by log10 (fold change). Fold change is calculated as the fraction of DEGs found in a pathway divided by the fraction of all expressed genes within the pathway. (E) Cell density pseudotemporal patterns of B cell subtype dynamics. The pseudotime of each cell was estimated using the Monocle algorithm. Colors indicate transcriptional states. (F) Expression level of different gene sets (adaptive immune response, innate immune response, and antigen presentation) across six‐time points in neutrophils. DPI: days post‐infection.

B cells play a vital role in adaptive immunity and antibody production in teleosts, particularly in the spleen and head kidney.^[^ ^36^ ^]^ Using scRNA‐seq, we identified nine distinct B cell subsets representing a spectrum of differentiation and functional states, including early (B‐ebf1), proliferating (B‐hmgb2a), naive (B‐ccnd2a, B‐mcsflr2), activated (B‐Ight, B‐Ighd, B‐tnfα, B‐jdp2b), and plasmocytes (B‐Ighm) (**Figure** **5**A,B). Notably, Ighm^+^ B cells were consistently elevated post‐infection, indicating a robust IgM‐mediated response, particularly in the early defense against pathogens (Figure 5C). GO analysis showed enrichment in antigen processing and presentation pathways, underscoring their central role in adaptive immunity (Figure 5D). Proliferative B cells were enriched in cell cycle genes, while tnfα^+^ B cells showed activation of innate immune‐related pathways. Pseudotime trajectory analysis revealed a clear differentiation path from early B cells to terminal Ighm^+^ plasmocytes (Figure 5E; Figure S5A,B, Supporting Information), with innate immune responses activated early and adaptive responses emerging later (Figure 5F). Together, these results provide a high‐resolution view of B cell functional dynamics and maturation in response to bacterial infection in fish.

### 2.6 Type II Interferon Signaling Regulates il8^+^ Neutrophils Activation in Response to Bacterial Infection

**Figure 6:**
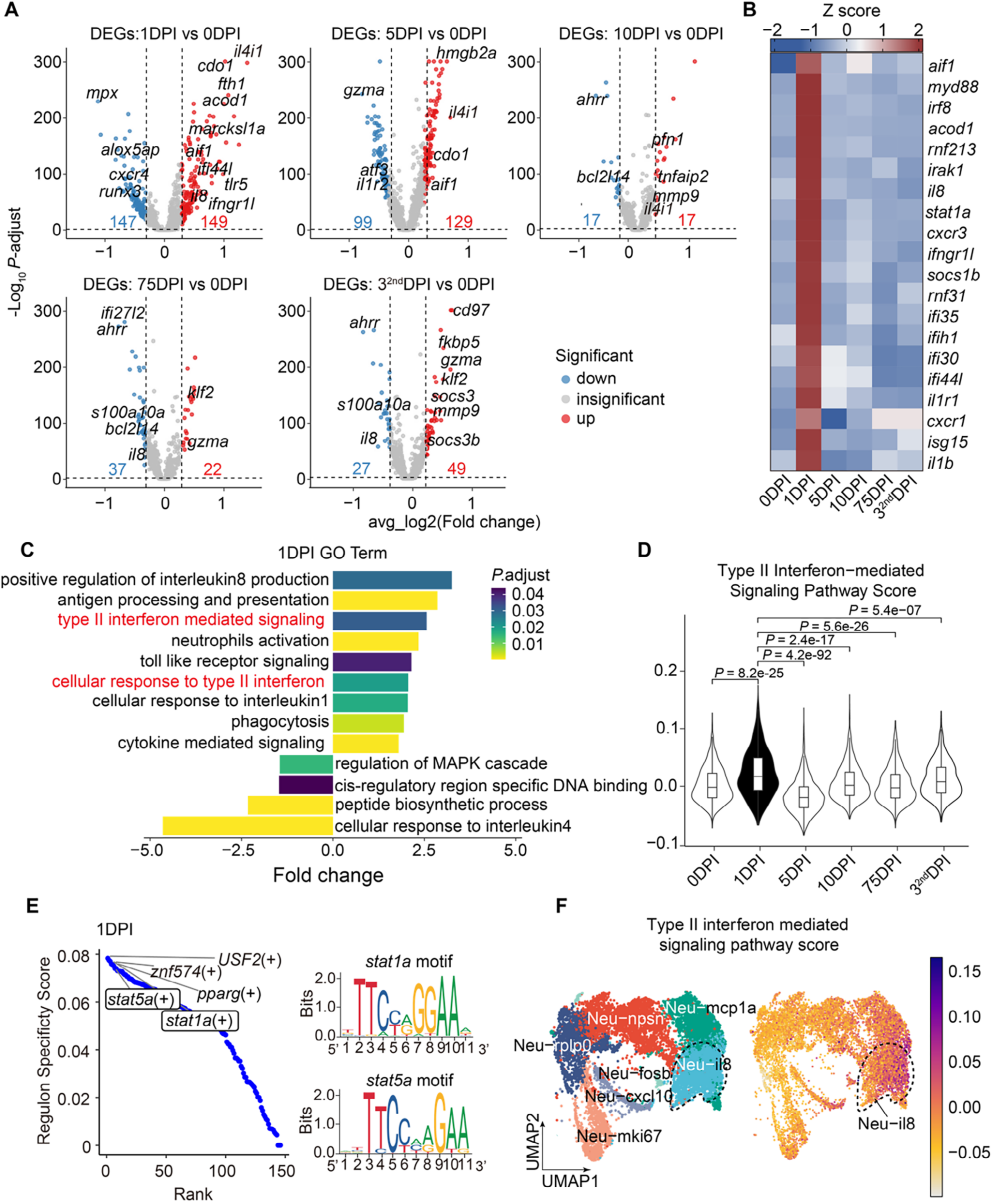
Type II interferon signaling pathway enriched in neutrophils for antibacterial responses to *S. agalactiae*. (A) Volcano plot showing differential gene expression for inflammatory genes at five‐time points post‐infection in neutrophils compared with the control group (0 DPI). DPI: days post‐infection. Genes significantly upregulated (red) or downregulated (blue) at each time point were identified by a two‐sided Wilcoxon rank‐sum test (Bonferroni‐adjusted *P*‐value < 0.05). DEGs: differentially expressed gene. DPI: days post‐infection. (B) Heatmap showing the expression of interferon‐related genes across six‐time points in neutrophils, with Z‐score normalized expression levels color‐coded. DPI: days post‐infection. (C) GO enrichment analysis of DEGs in neutrophils at 1 DPI. The right side shows significantly enriched GO terms, and the left side shows significantly depleted GO terms. The significance of enrichment is determined by a hypergeometric test. *P*‐value was adjusted by FDR method. DPI: days post‐infection. (D) Violin plot of type II interferon‐mediated signaling pathway scores (GO:00 60333) for neutrophils at six‐time points. For the box plot within each violin plot, middle lines indicate median values, boxes range from the 25th to 75th percentiles, and upper/lower whiskers extend from the hinge to the largest/smallest value no further than 1.5 times the interquartile range (IQR) from the hinge. Significance was determined by a two‐sided Wilcoxon rank‐sum test. DPI: days post‐infection. (E) Cell type specificity of inferred TFs by SCENIC. Regulon Specificity Score (range from 0 to 1) was estimated to order the TFs in neutrophils at 1 DPI. The top5 of TFs were highlighted, with binding motifs of *stat1a* and *stat5* shown on the right. DPI: days post‐infection. (F) UMAP visualization of neutrophil subsets (left) and the expression pattern of genes involved in the type II interferon‐mediated signaling pathway (GO:00 60333) (right) in neutrophils, Neu‐il8 subset was circled by the dashed box, Colors represent the expression of genes in each cluster.

Neutrophils are key players in the rapid defense against pathogens, with their functions well‐studied in mammals.^[^ ^24^, ^37^, ^38^ ^]^ However, their antibacterial roles in teleosts remain less understood. We analyzed differential gene expression in neutrophils at various time points post‐infection (**Figure** **6**A) and observed a significant upregulation of inflammatory genes at 1 DPI, marking this as a critical time point for the neutrophil response to bacterial infection (Figure 6A). Many of these upregulated genes were linked to the interferon signaling pathway, with a marked increase in interferon‐related genes at 1 DPI (Figure 6B). Among them, *rnf123*, known for its bactericidal function in human cells,^[^ ^39^ ^]^ suggests a conserved role of the interferon pathway in neutrophil‐mediated immunity. Additionally, GO enrichment analysis (Figure 6C) and gene module scoring (Figure 6D) revealed significant enrichment of the type II interferon signaling pathway during this period, highlighting its critical role in neutrophil responses to bacterial infection.

To further investigate gene regulatory networks at different time points, we employed single‐cell regulatory network inference and clustering (SCENIC) analysis^[^ ^40^ ^]^ to identify regulons (transcription factors, TFs) activity with significant *cis‐*regulatory motif enrichment in neutrophils across different time points. SCENIC analysis revealed that the regulon activity of TFs involved in the type II interferon signaling pathway, such as *stat1a* and *stat5*, were specifically detected at 1 DPI (Figure 6E).

To examine the relationship between type II interferon signaling and neutrophil subsets, we calculated signaling scores across all neutrophil subsets. The highest scores were observed in il8^+^ neutrophils (Figure 6F), suggesting that type II interferon signaling is likely involved in regulating il8^+^ neutrophil activation during bacterial infection.

### 2.7 Conserved IFNγ‐Induced State Transition to il8^+^ Neutrophils in Nile Tilapia and Human

**Figure 7:**
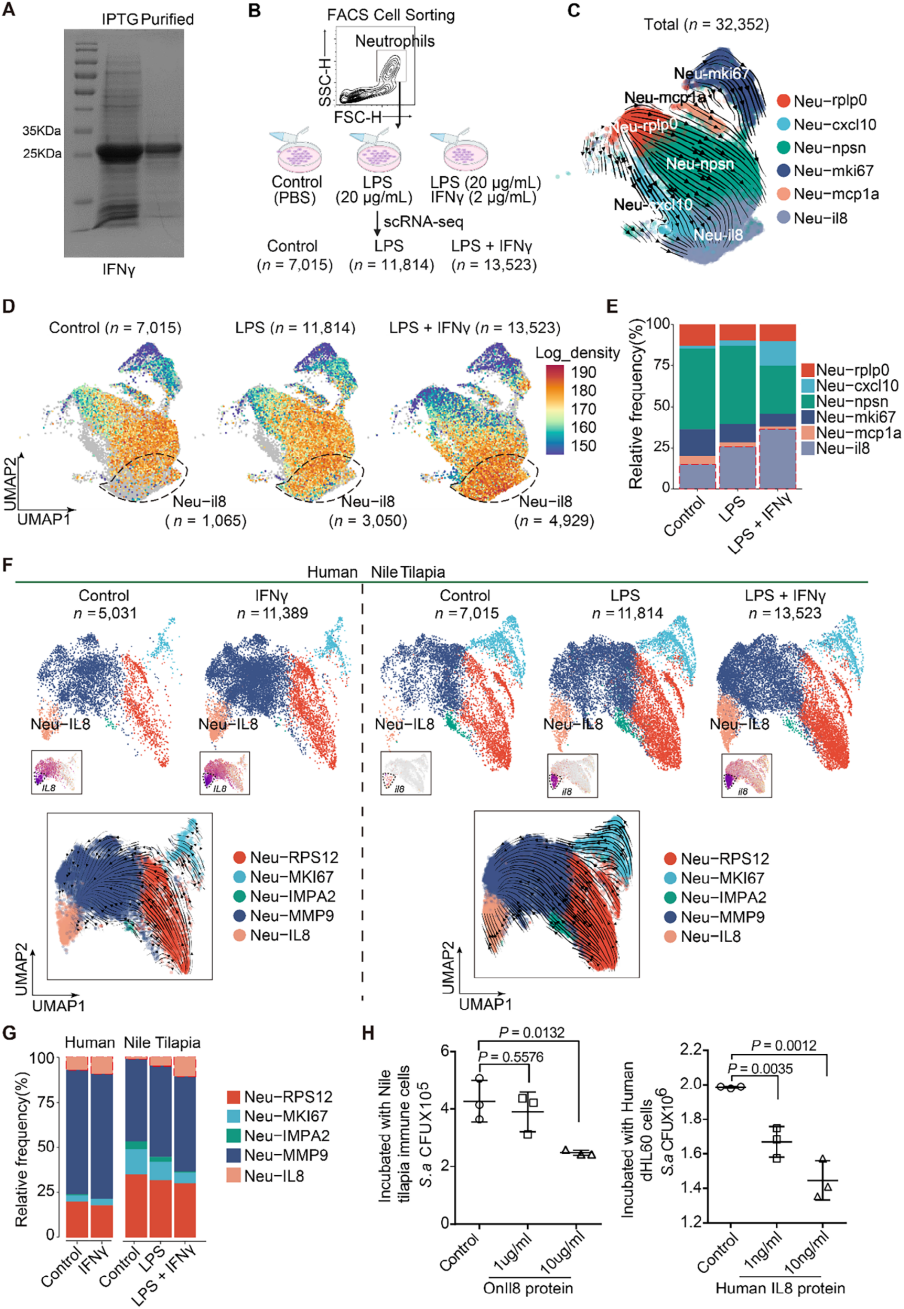
IFNγ signaling primes neutrophils state transition to il8^+^ subtype with antibacterial effect in teleost and mammals. (A) SDS‐PAGE assay showing the purification of recombinant tilapia IFNγ protein in *Escherichia coli*. The lanes from left to right represent protein markers, unpurified IFNγ protein induced by IPTG, and purified IFNγ protein. (B) Gated neutrophils by flow cytometry from tilapia (*n* = 3–4), plated in replicates and cultured under unstimulated, LPS‐stimulated, or LPS + IFNγ‐stimulated conditions for 4 h, followed by scRNA‐seq. After quality control, 7,015 cells from control, 11,814 cells from LPS‐stimulated, and 13,523 cells from LPS + IFNγ‐stimulated conditions were retained. (C) UMAP visualization of neutrophil subtype transitions by RNA velocity analyses. Arrows indicate the directionality of the cell‐cell transition matrix computed by CellRank. Cells are color‐coded by subtypes. A total of 32,352 cells from three groups, including 7,015 cells from control, 11,814 cells from LPS‐stimulated, and 13,523 cells from LPS + IFNγ‐stimulated conditions were analyzed. (D) UMAP visualization of Neu‐il8 subtype density by Mellon analysis across control, LPS‐stimulated, and LPS + IFNγ‐stimulated groups. Neu‐il8 subset was circled by the dashed boxes. UMAPs colored by Mellon log density. Density was computed in high‐dimensional cell‐state space (diffusion maps) (E) Bar plot showing the proportions of neutrophil subtypes across unstimulated, LPS‐stimulated, and LPS + IFNγ‐stimulated conditions. Red boxes highlight Neu‐il8 subset. Cell subtypes are color‐coded. (F) UMAP visualization of neutrophils (top panel) overlaid with RNA velocity maps (bottom panel) in humans (unstimulated, IFNγ‐stimulated) and Nile tilapia (unstimulated, LPS‐stimulated, and LPS + IFNγ‐stimulated), analyzed independently. The expression level of IL8 is highlighted in the left‐bottom corner. Arrows indicate the directionality of the cell‐cell transition matrix computed by CellRank. Cell types are color‐coded. (G) Bar plot showing the proportions of neutrophil subtypes in humans and tilapia. Cell types are color‐coded. (H) Scatter plot showing the *S. agalactiae* CFU recovered from control and Il8 ‐stimulated immune cells (left), and from control and IL8 ‐stimulated dHL‐60 cells (right) (Methods). *S. agalactiae* was incubated with control and Il8 ‐stimulated immune cells for 5 h, and then plated on BHI agar for CFUs enumeration.

To confirm the role of IFNγ signaling, we developed a recombinant tilapia IFNγ protein and used it to stimulate primary immune cells (**Figure** **7**A). Surface binding was verified using His‐tagged IFNγ and FACS with anti‐His antibodies (Figure S6A, Supporting Information), and qPCR showed significant IL8 upregulation (Figure S6B, Supporting Information). To further explore the IFNγ‐IL8 axis, we cultured neutrophils with LPS alone or combined with IFNγ (Figure 7B), followed by scRNA‐seq. This yielded 32,352 high‐quality cells clustered into six neutrophil subtypes based on known markers (Figure 7C; Figure S6C–E, Supporting Information).

Velocity analysis revealed that neutrophil differentiation progressed from rplp0^+^ neutrophils to il8^+^ neutrophils, a trajectory consistent with findings from Monocle and CellRank analyses (Figure 7C). Diffusion maps and Mellon analysis indicated a shift in cell‐state density toward il8^+^ neutrophils following IFNγ stimulation, suggesting that these cells became the predominant subtype (Figure 7D). This was further supported by a significant increase in the proportion of il8^+^ neutrophils after IFNγ stimulation (Figure 7E). Gene set enrichment analysis (GSEA) confirmed that the type II interferon signaling pathway was significantly enriched in il8^+^ neutrophils after IFNγ stimulation, indicating its activation (Figure S6F, Supporting Information). Moreover, integrating IFNγ‐stimulated human and tilapia neutrophil datasets revealed five clusters based on marker genes (Figure S6G,H, Supporting Information). Both human and Nile tilapia showed a substantial expansion of il8⁺ neutrophils following IFNγ stimulation (Figure 7F,G), indicating that IFNγ signaling conservedly promotes il8⁺ neutrophils differentiation across species.To directly compare IFNγ responses across species, we included an IFNγ‐only treatment group in tilapia, which significantly upregulated IL8 expression (Figure S6I,J, Supporting Information). Co‐stimulation with IFNγ and LPS further enhanced this effect, indicating an additive response and reinforcing the conserved role of IFNγ‐IL8 signaling in neutrophil activation.

To assess the evolutionary conservation of IL8 signaling, we produced recombinant tilapia IL8 and found it significantly enhanced bacterial killing by tilapia neutrophils(Figure 7H; Figure S6K, Supporting Information). Similarly, human IL8 increased the antibacterial capacity of differentiated HL‐60 cells (Figure 7H), supporting a conserved IL8 signaling function. Blocking the IL8 pathway with the CXCR1/2 inhibitor reparixin^[^ ^41^ ^]^ significantly reduced antibacterial activity (Figure S6L, Supporting Information), further confirming its functional relevance. Additionally, tilapia IL8 enhanced antibacterial function in human HL‐60 cells (Figure S6M, Supporting Information).

Mechanistically, transcription factor analysis identified Stat1 as a key mediator of IFNγ signaling. IFNγ pathway inhibition^[^ ^42^ ^]^ reduced STAT1/p‐STAT1 levels and suppressed IL8 transcription (Figure S7A–F, Supporting Information). Downstream, IL8 stimulation increased both phagocytic uptake of bacteria and intracellular ROS production in tilapia and human neutrophils (Figure S7G–L, Supporting Information), indicating that IL8 promotes antibacterial defense through enhanced phagocytosis and ROS‐dependent killing.

## 3 Discussion

### 3.1 Single‐Cell Atlas of Immune Cell Dynamics in Nile Tilapia in Response to *S. agalactiae* Infection

Despite several scRNA‐seq studies on teleosts,^[^ ^16^, ^17^, ^18^, ^19^, ^43^, ^44^ ^]^ a comprehensive analysis of immune cell dynamics following bacterial infection has been lacking. In this study, we present what is likely the first detailed immune landscape of tilapia, an emerging model species of increasing importance for both basic research and its economic value in aquaculture, generated by analyzing high‐throughput single‐cell transcriptomic profiles of >113 K immune cells over an extended period following *S. agalactiae* infection. Coupled with fluorescence‐activated cell sorting assays, we identified major immune cell populations and their distinct subsets based on unique molecular signatures throughout the infection process. Our focus was on neutrophils, which play a crucial role in the early inflammatory response. We found that IFNγ signaling is significantly enriched in the il8^+^ neutrophil subset, driving the transition of neutrophils to an inflammatory state, consistent with findings in human neutrophils. These results establish a reference model and framework for studying immune cell functional diversity, dynamics, and time‐specific molecular signatures during bacterial infection in vertebrates, while also suggesting potential neutrophil‐based therapeutic strategies for bacterial diseases in both fish and humans. Additionally, we provide a valuable resource for the research community, with the dataset accessible via a user‐friendly portal at https://hulabshou.shinyapps.io/tilapia/.

### 3.2 The Dynamics of Macrophages, T cells, and B cells During *S. agalactiae* Infection

Our data reveal distinct, time‐dependent dynamics across immune cell types. Neutrophils and macrophages act as early responders, contributing to pathogen clearance via phagocytosis, antigen presentation, and secretion of inflammatory mediators. Subsequently, T and B cells drive adaptive immune responses through cytokine signaling and antibody production. In addition, ligand‐receptor interaction analysis revealed significant intercellular communication among these populations, supporting a coordinated innate‐to‐adaptive immune transition. This stepwise activation reflects a conserved immune architecture across vertebrates, while also highlighting species‐specific features in fish immunity compared to mammals. These insights strengthen the relevance of *S. agalactiae*‐infected tilapia as a model to understand fundamental and comparative immunology.

### 3.3 IFNγ‐STAT1‐IL8 Axis and Neutrophil Plasticity in Teleosts

While IFNγ is known to promote macrophage polarization and Th1 responses in mammals,^[^ ^45^, ^46^ ^]^ its role in neutrophil plasticity in fish remains underexplored. Our data reveal that IFNγ signaling activates Stat1a in tilapia neutrophils, as evidenced by increased *stat1a* expression and Stat1 motif enrichment at 1 DPI. Inhibition of STAT1 reduced both total and phosphorylated STAT1 levels and significantly suppressed *IL8*transcription, indicating that IL8 induction is STAT1‐dependent.

Cross‐species scRNA‐seq integration confirmed that IFNγ promotes the expansion of il8^+^ neutrophils in both tilapia and humans, suggesting a conserved IFNγ‐STAT1‐IL8 regulatory axis. Functionally, IL8 stimulation enhanced phagocytosis and ROS production in neutrophils, highlighting its effector role in antibacterial responses.

In mammals, IFNγ drives N1‐like neutrophil polarization with antimicrobial activity, and IL8^+^ subset playing a key role in the inflammatory response.^[^ ^24^, ^26^, ^27^, ^28^ ^]^ Our findings indicate that a similar IFNγ‐driven pro‐inflammatory state (il8^+^ neutrophils) emerges in teleosts, supporting the existence of conserved neutrophil activation trajectories. Unlike mice and zebrafish, tilapia possess IL8 homologs and naturally exhibit GBS susceptibility and human‐like immune symptoms, making them a valuable model for neutrophil biology.

Functionally, recombinant tilapia IL8 enhanced bacterial killing in both tilapia and human neutrophil‐like cells (dHL‐60). Importantly, inhibition of CXCR1/2 by reparixin impaired neutrophil antimicrobial function, confirming the functional relevance of IL8‐CXCR1/2 signaling. Overall, our results establish a conserved IFNγ‐STAT1‐IL8 axis that drives neutrophil activation during bacterial infection and underscore the value of tilapia as a model to study conserved innate immune mechanisms. Further research is needed to define STAT1 downstream targets and to assess the therapeutic potential of this axis across species.

Despite the strengths of our study, several limitations remain. Although our findings support the regulatory and functional conservation of the IFNγ‐IL8 axis, cytokine activity is not always cross‐compatible across species due to receptor‐ligand mismatches. This underscores the need for species‐matched reagents when studying cytokine signaling in evolutionarily distant organisms. Moreover, the lack of validated antibodies and gene‐editing tools, such as IFNγ receptor knockouts in Nile tilapia, hinders more comprehensive mechanistic investigations. Our functional validation relied primarily on ROS and phagocytosis assays; future studies should incorporate NETosis assays to better assess neutrophil‐mediated antibacterial functions. While the IFNγ‐IL8 axis shows therapeutic promise, its translational potential remains to be tested. Future work should aim to develop genetic tools for tilapia, expand functional assays, and perform translational studies in mammalian infection models to evaluate efficacy, safety, and patient stratification. Addressing these gaps will be crucial to fully elucidate the IFNγ‐IL8 axis and its role in host defense.

Our single‐cell profiling of Nile tilapia immune cells reveals diverse subsets and dynamic responses during *S. agalactiae* infection. We identify a conserved IFNγ‐IL8 signaling pathway that drives the activation of il8⁺ neutrophils, providing new insight into vertebrate antibacterial immunity. This transition may have been missed in prior models due to limited resolution or alternate immune stimuli (e.g., LPS, TNFα). Leveraging the natural susceptibility of tilapia enabled detection of this conserved mechanism, reinforcing the value of alternative fish models in uncovering fundamental immune processes and informing neutrophil‐targeted therapeutic strategies.

## 4 Experimental Section

### Experimental Animals

Nile tilapia (*Oreochromis niloticus*) were maintained in a recirculating water system at 28 °C and fed daily prior to the start of experiments. Healthy fish, ≈8–10 cm in length, were selected for the experiments. All procedures were conducted in accordance with the guidelines of the Committee on Laboratory Animal Care and Use of Shanghai Ocean University under protocol # SHOU‐DW‐2021‐068.

### Bacterial Infection

*S. agalactiae* was cultured in Brain‐Heart Infusion (BHI) Broth (Hopebio, HB8297‐5) until reaching the exponential growth phase. The bacteria were then harvested, washed, and resuspended in PBS (Sangon Biotech, E607008). Fish were infected with a bacterial suspension at a semi‐lethal concentration (2 × 10⁷ CFU/mL). Nile tilapia (15 ± 2.5 g, 10.0 ± 1.4 cm) were challenged via intraperitoneal injection with 50 µL of the bacterial suspension, while control fish were injected with 50 µL of PBS (Sangon Biotech, E607008). During the infection process, 19 fish were used for infection, and 18 fish were left after infection. The fish were then maintained until sampling at 1, 5, 10, and 75 DPI. Surviving fish at 75 DPI were immediately re‐infected with the same bacterial concentration via intraperitoneal injection, and samples were collected at 3 days post‐reinfection. At each time point, single‐cell suspensions were prepared from three individual fish, pooled in equal proportions, and processed for single‐cell RNA library construction.

### Leukocyte Isolation

Leukocytes were isolated from the head kidney and spleen of control and infected Nile tilapia using a 51/34% discontinuous Percoll (GE Healthcare, 17 089 109) density gradient, following previously described protocols.^[^ ^47^, ^48^ ^]^ Briefly, the tissues were mechanically dissociated to obtain a cell suspension in L‐15 medium (Gibco, 41 300 039) supplemented with 5% fetal bovine serum (FBS) (Gibco, A3161002C), which was then layered over 51/34% discontinuous Percoll gradients. After centrifugation at 400 g for 30 min, leukocytes located at the gradient interface were collected and washed with L‐15 medium for further use.

### Flow Cytometry and BrdU Incorporation

To identify CD3^+^ T cell populations, the anti‐tilapia CD3 monoclonal antibody (mAb) was developed as previously described.^[^ ^49^ ^]^ Tilapia leukocytes were isolated and stained with FITC‐conjugated CD3ε mAb and live/dead‐violet dye. Incubations with primary and secondary antibodies were performed on ice for 30 min, followed by two washes with PBS (Sangon Biotech, E607009‐0500) containing 2% FBS (Gibco, A3161002C) after each incubation. For the BrdU incorporation assay, control or *S. agalactiae*‐infected Nile tilapia were intraperitoneally injected with 0.75 mg BrdU (Sigma–Aldrich, 19–160) in 200 µL PBS 1 d before sacrifice. Spleen leukocytes were isolated for 24 h. A total of 2 × 10^6^ spleen leukocytes were first stained for surface CD3ε as described above. The cells were then fixed with BD Cytofix/Cytoperm Buffer (BD Bioscience, 554 722) on ice for 30 min and washed twice with BD Perm/Wash Buffer (BD Bioscience, 554 723). Subsequently, the cells were treated with BD Cytoperm Plus Buffer (BD Bioscience, 561 651) for 10 min and BD Cytofix/Cytoperm Buffer for 5 min on ice, followed by digestion with 300 µg/mL DNase at 37 °C for 1 h. After washing with BD Perm/Wash Buffer, the samples were stained with 1:100 diluted FITC‐anti‐BrdU antibody (BD Bioscience, 51–33284X) at room temperature for 20 min and analyzed by flow cytometry.

### Phagocytosis Assay

1 × 10^9^ CFU *S. agalactiae* was inactivated in 4% paraformaldehyde at 4 °C for 24 h. The inactivated *S. agalactiae* was incubated with 0.02 g mL^−1^ FITC (Sigma–Aldrich, F3651 A) in 0.1 M NaHCO_3_ at 37 °C for 30 min. To assess the opsonization function of myeloid cells, 2 × 10^7^ CFU FITC‐labeled *S. agalactiae* were washed with PBS and added to a suspension of 5 × 10^6^ head kidney leukocytes. The mixture was incubated at 28 °C for 6 h, after which phagocytosis by myeloid cells was analyzed using flow cytometry. Myeloid cells were gated by flow cytometry according to a previously described method.

### Recombinant Protein Preparation

The coding sequencing for Nile tilapia IFNγ(excluding the signal peptide) were ligated into the pET‐28a (+) vector as previously reported.^[^ ^50^ ^]^ Recombinant plasmids were transformed into Transetta (DE3) chemically competent cells (TransGen Biotech, CD80102), and protein expression was induced with 0.5 mM IPTG at 37 °C for 4 h. The unpurified protein was re‐folded by gradient dialysis at 4 °C. Recombinant IFNγ, tagged with His, was purified using Ni‐NTA affinity chromatography (Cytiva, 17 524 801). The purified proteins were respectively concentrated using Microcon‐10 kDa and Microcon‐3 kDa molecular mass centrifugal filter units (Millipore, UFC901096‐24), quantified using a BCA Protein Assay Kit (Thermo Fisher, 23 227), and stored at −80 °C until use. For Il8 protein expression and purification, The codon‐optimized mature peptide sequence was cloned into pET32a via NcoI/HindIII sites and expressed in Rosetta‐gami (DE3) pLysS cells. Induction was performed at OD600 0.4–0.6 with 0.5 mM IPTG (37 °C, 6–8 h). Cells were lysed by pressure homogenization, and the clarified lysate was purified by Ni‐affinity chromatography (elution: 20 mM Tris‐HCl, pH 6.8, 500 mM imidazole, 100 mM NaCl). Further purification included size‐exclusion and anion‐exchange chromatography (AEX; 20 mM Tris‐HCl, pH 6.8, with 0.1–1 M NaCl gradient). The fusion protein was buffer‐exchanged (20 mM Tris‐HCl, pH 6.8, 100 mM NaCl) and digested with enterokinase (16 °C, 24 h). Due to the target protein's pI (7.907), it was recovered in the AEX flow‐through. SDS‐PAGE (Figure S6K, Supporting Information) confirmed purification success.

### HL‐60 Culture Under DMSO Exposure

HL‐60 cells (Pricella, CL0110) were cultured in RPMI 1640 (Gibco,11 875 093) containing with 10% FBS (Gibco, A3161002C) at 37 °C in 5% CO_2_. Cell cultures were passaged two to three times per week to maintain a density of 1–2 × 10^6^ cells/mL. For passaging, cells were collected by centrifugation (100 x g, 4 min) and then resuspended in 8 mL of pre‐warmed RPMI 1640 medium (Gibco,11 875 093) containing 10% FBS (Gibco, A3161002C) and transferred to cell culture dishes (Corning, 430 167). To obtain differentiated HL‐60 neutrophil‐like cells (dHL‐60), HL‐60 cells were treated with RPMI 1640 (Gibco,11 875 093) containing with 10% FBS (Gibco, A3161002C) and 1.25% (v/v) DMSO (Sigma–Aldrich, D5879) for 6 d at 37 °C in 5% CO_2_.^[^ ^51^ ^]^

### Immune Cells‐Bacteria Cultures

Tilapia leukocytes were seeded at a density of 4 × 10^5^ cells per well in round‐bottom 48‐well plates containing DMEM/RPMI1640 medium (Gibco, C11995500BT, 11 875 093) supplemented with 10% FBS (Gibco, A3161002C) and 1 µg mL^−1^ and 10 µg mL^−1^ purified Il8 protein. Similarly, dHL‐60 cells were treated with RPMI 1640 medium (Gibco,11 875 093) containing 10% FBS (Gibco, A3161002C) with 1 and 10 ng mL^−1^ purified IL8 protein (R&D systems, 208‐IL‐010). After a 24 h‐incubation, for the bacterial killing assay, cells were washed with PBS (Sangon Biotech, E607008), and opsonized *S. agalactiae* was added at a multiplicity of infection (MOI) of 1–10 in a total volume of 200 µL. The plates were incubated at 37 °C for 5 h. A 20 µL aliquot of the culture (10% of the total volume) was taken and treated with 980 µL 0.25% Triton X‐100 (Sangon Biotech, A110694‐0500) to lyse cells at 37 °C for 20 min. A 10 µL aliquot of the lysate was then serially diluted and plated on BHI agar (Hopebio, HB8478) for overnight incubation to enumerate CFUs.

### Isolation and Cell Multiplexing Oligos Labeling of Cells

Leukocytes were isolated from the head kidney and spleen of control and infected Nile tilapia. For scRNA‐seq library preparation, 0.5–2 × 10^6^ cells were transferred to 2 mL microcentrifuge tubes and centrifuged at 400 g for 5 min using a swinging‐bucket rotor (Eppendorf 5810R). The supernatant was removed, and the cells were resuspended in 100 µL of Cell Multiplexing Oligos (CMOs, 10x Genomics, PN‐1000261) and incubated for 5 min at room temperature. Following CMO labeling, cells were maintained at 4 °C and washed three times with chilled DPBS (Gibco, C14190500BT) containing 10% FBS to remove unbound CMOs. The labeled cells were then resuspended in 500 µL of ice‐cold PBS containing 0.04% bovine serum albumin (BSA, Sigma–Aldrich, A8806) and pooled for scRNA‐seq. Cell viability and concentration were checked and confirmed to be above 90% and 1,300–1,600 cells/µL, respectively.

### scRNA‐seq Library Preparation and Sequencing

For high‐quality scRNA‐seq data, we utilized the Chromium Next GEM Single Cell 3′ Reagent Kits v3.1 with Feature Barcode technology for Cell Multiplexing (10x Genomics, CG000388). All assays were performed with a targeted recovery of 30,000 cells, which were loaded onto the Chromium Controller following the manufacturer's instructions. Briefly, cells were pooled and loaded onto a 10x Genomics chip to generate Gel Beads‐in‐Emulsion (GEMs). Within each GEM, polyadenylated mRNA was captured, and cDNA was generated from single cells. The cDNA was then amplified and purified using 0.6X SPRISelect beads (Beckman Coulter, B23318). Gene expression (GEX) libraries were constructed from the cDNA through a series of steps: fragmentation, end repair, A‐tailing, double‐sided size selection, adaptor ligation, and sample index PCR. For cell multiplexing library construction, DNA from the CMO Feature Barcode (supernatant) was indexed. Libraries were quantified using the Qubit dsDNA HS Assay Kit (Invitrogen, Q32854) and Agilent 2100 Bioanalyzer High Sensitivity DNA Kit (Agilent Technologies, 50 674 626). Finally, the GEX and cell multiplexing libraries were pooled at a 4:1 ratio and sequenced on an Illumina NovaSeq 6000 S4 PE150 (Read 1: 28 cycles, i7 index: 10 cycles, i5 index: 10 cycles, Read 2: 90 cycles).

### Neutrophils Culture and IFNγ Protein Stimulation

Neutrophils were cultured as previously described.^[^ ^27^ ^]^ Briefly, leukocytes were isolated from the head kidney and spleen of healthy Nile tilapia using a 51/34% discontinuous Percoll (GE Healthcare, 17 089 109) density gradient. Neutrophils were then sorted by flow cytometry (BD FACS Melody) according to cell size and granularity as described in prior studies.^[^ ^52^ ^]^ The sorted neutrophils were seeded at a density of 2.5 × 10^5^ cells per well in round‐bottom 48‐well plates containing DMEM/RPMI1640 medium (Gibco, C11995500BT, 11 875 093) supplemented with 10% FBS (Gibco, A3161002C), 100 µg mL^−1^ streptomycin, and 100 U/mL penicillin (Gibco, 15 140 122). The medium was further supplemented with either LPS (20 µg mL^−1^) alone or LPS (20 µg mL^−1^) in combination with IFNγ (2 µg mL^−1^). The cell suspension was incubated at 28 °C with 5% CO_2_ for 4 h. Cell viability was assessed using 0.4% Trypan Blue (Gibco, 15 250 061) and counted manually with a hemocytometer (Incyto, DHC‐F015) (≥90% viability and 700–1200 cells/µL concentration required). The single‐cell transcriptome library was then generated using the Chromium Next GEM Single Cell 3ʹ Reagent Kits v3.1 (10x Genomics, CG000204).

### qPCR

Total RNA was isolated from cells using TRIzol® reagent (Invitrogen, 15596018CN) following the manufacturer's instructions. RNA (10 ng‐1 µg) was reverse‐transcribed into cDNA using ABScript Neo RT Master Mix for qPCR with gDNA Remover (Abclonal, RK20433) in a 20 µL reaction. qPCR was performed using SsoFast Eva GreenSupermix (BIO‐RAD, 1 725 201). Melt curve analysis (60–95 °C) confirmed primer specificity. Relative gene expression was calculated by the 2−ΔΔCt method, normalized to β‐actin, and expressed as fold change versus the control group. All reactions were run in triplicate.

### Western Blot

Cells were lysed in RIPA buffer (Beyotime, P0013B) containing 1× protease/phosphatase inhibitors (Thermo, 78 420) on ice for 30 min, followed by centrifugation 12,000 × g, 10 min, 4 °C to remove debris. Total protein concentration was determined using a Bradford/BCA assay kit (Thermo, 23 227), with bovine serum albumin (BSA) as the standard. Protein samples (20–50 µg per lane) were mixed with 4× SDS‐PAGE loading buffer (Solarbio, P1016), denatured at 95 °C for 5 min, and resolved on 8% SDS‐polyacrylamide gels at 80–120 V for 1.5–2 h. Proteins were transferred onto PVDF membranes (MerckMillipore, IPVH00010) using a wet transfer system. Membranes were blocked with 5% non‐fat milk (Seven, SW128‐03) in TBST (Tris‐buffered saline with 0.1% Tween‐20) for 1 h at room temperature, then incubated with primary antibodies (STAT1: CST, 9172S, P‐STAT1: CST, 8009)diluted in blocking buffer overnight at 4 °C. After washing (3 × 10 min in TBST), membranes were incubated with HRP‐conjugated secondary antibodies (Abbkine, A25222) (1:4000) for 1 h at RT. Signals were developed using ECL substrate (Abbkine, BMU102‐CN) and captured by a chemiluminescence imaging system.

### ROS Detection

Cells were harvested and washed twice with PBS. Cells (1 × 10⁶ cells/mL) were incubated with a fluorescent ROS‐sensitive probe ( DCFH‐DA)(Solarbio, CA1410) for 20 min at 37 °C in the dark. After incubation, cells were washed twice with PBS to remove excess probe. Cells were resuspended in PBS and analyzed immediately using a BD Melody flow cytometer. Quantification of mean fluorescence intensity (MFI) using FlowJo software.

### Inhibitor Treatment

For IL8 inhibitor assay, dHL‐60 cells were first treated with 100 µm CXCR1/2 inhibitor reparixin (IL8 pathway blocker)^[^ ^41^ ^]^ (MedChemExpress, HY‐15251) at 37 °C for 24 h. For STAT1 inhibitor assay, dHL‐60 cells were first stimulated with 200 ng ml^−1^ IFNγ protein (MedChemExpress, HY‐P70610G) and 200 µm Fludarabine^[^ ^42^ ^]^ (MedChemExpress, HY‐B0069) at 37 °C for 24 h.

### Flow Cytometer and p‐STAT1

For intracellular p‐STAT1 staining, dHL‐60 cells were first stimulated with 200 ng ml^−1^ IFNγ protein (MedChemExpress, HY‐P70610G) and 200 µm Fludarabine (MedChemExpress, HY‐B0069) for 24 h and then fixed with the fixation/permeabilization solution (BD Biosciences, 554 722) on ice for 30 min. These cells were then washed with the Perm/Wash solution (BD Biosciences, 554 723) twice and further stained with 1:200 diluted Alexa Fluor 647‐conjugated anti‐p‐STAT1 Tyr701 (CST, 8009) on ice for 30 min. All thestained cells were resuspended with FACS buffer and analyzed by BD FACSCanto II flow cytometer. Data were analyzed using FlowJo software.

### Antibodies

For Figures 1I and 4D, a tilapia‐specific CD3 antibody validated in our previous publications^[^ ^49^ ^]^ was employed. In Figures 3G and 4D, a commercially available BrdU antibody (BD Bioscience, 51–33284X) was used for detecting BrdU^+^ T cells in tilapia. For Figure S6A (Supporting Information), an anti‐His antibody (BBI, D191001‐0100) was used to detect His‐tagged recombinant IFNγ protein. For Figure S7D,E, (Supporting Information), commercial STAT1 (CST, 9172S) and p‐STAT1 antibodies (CST, 8009) were used.

### scRNA‐Seq Data Analysis—Data Processing

Sequences from each sample were processed using CellRanger's multi‐pipeline with default parameters (10x Genomics, version 7.1.0). This pipeline performs alignment based on the *Oreochromis niloticus* reference genome (Ensembl 108, GCA_0 018 58045.3), followed by filtering, barcode counting, and UMI counting. The resulting UMI count matrix was analyzed with the R package Seurat (version 4.2.0).^[^ ^53^ ^]^ Doublet removal was conducted using the Python package Scrublet (version 0.2.3).^[^ ^54^ ^]^ Genes expressed in fewer than three cells were excluded. Cell quality was assessed using three metrics: (1) total UMI count per cell below 30,000; (2) number of detected genes between 200 and 4,000; and (3) mitochondrial gene percentage below 15%. Thresholds were manually determined based on the distribution of each quality control metric, and cells not meeting these criteria were discarded.

### Batch Correction and Dimensionality Reduction

Variability between cells from different samples was observed, likely due to biological and technical differences. While batch effect minimally affected the overall partitioning of cell types, clustering within specific cell types showed a sample‐based pattern. To address this, we employed SCTransform (parameters: method = “glmGamPoi”, vars. to. regress = c (“percent. mt”), vst. flavor = “v2”) to correctly for sequencing depth and mitochondrial gene expression. The top 3,000 highly variable genes identified were then used for PCA, with RunPCA (parameter: npcs = 30) to calculate PCA embeddings. RunUMAP function was subsequently used for 2D cell visualization. For single immune cell subtype analysis (Figure 1B), Harmony (version 0.3.5)^[^ ^55^ ^]^ was employed to integrate cells from different sample IDs, providing corrected PCA for subsequent clustering and UMAP visualization.

### Unsupervised Clustering and Annotation

For Figure 1B, the FindClusters function, based on the unsupervised Louvain algorithm, was used for cell clustering. To determine the optimal resolution, we tested multiple values (0.1, 0.2, 0.6, 0.8, and 1.0) and performed post‐hoc cluster comparisons to evaluate clustering performance. We selected 0.2 as it offered the best balance between avoiding over‐clustering and maintaining biological interpretability. At this resolution, clusters exhibited distinct transcriptional signatures and captured detailed heterogeneity. Cell subtypes were accurately annotated by identifying marker genes for each cluster through DEGs, obtained using the FindAllMarkers function (test. use = “wilcox”, adjusted *P*‐value < 0.05).

### Data Quality Assessment

To assess the quality of our data, we downloaded published scRNA‐seq data of Nile tilapia head kidney.^[^ ^16^ ^]^ The sequences from the sample were processed using CellRanger's pipeline with default parameters (10x Genomics, version 7.1.0). Subsequently, we employed the FindTransferAnchors function from Seurat to identify anchors between the published data and our head kidney data. After identifying the anchors, we utilized the MapQuery function to classify the query cells based on reference data (head kidney 0 DPI), consisting of a vector of reference cell type labels, and to facilitate the projection of the query onto the reference UMAP structure. Afterward, we used the DimPlot function for UMAP visualization and the VlnPlot function to compare the UMI counts and gene numbers in our data with those of published datasets (Figure S2B–D, Supporting Information).

### Identification of DEGs

For Figure 6A and Figure S3A, Supporting Information), DEGs between different time points post‐bacterial infection (1, 5, 10, 75 DPI, and 3 days post‐reinfection) and control (0 DPI) were identified using the FindMarkers function in Seurat, based on normalized data and applying a two‐sided Wilcoxon rank‐sum test. The *P*‐values were adjusted using the Bonferroni correction. DEGs were identified using conservative thresholds of |log2FC| > 0.3 and p.adjust < 0.05 to ensure both statistical rigor and biological relevance.

### Enrichment Analysis

To identify functional categories associated with specific gene lists, GO annotations were obtained by aligning Nile tilapia protein sequences against the EggNOG database using EggNOG‐mapper (version 2.1.9).^[^ ^56^ ^]^ Enrichment analysis was performed using a hypergeometric test, with the *P*‐value and fold change calculated using the following formulas:

(1) $$p=1-\sum_{i=0}^{m-1}\frac{\left(\begin{array}{c} M\\ i \end{array}\right)\left(\begin{array}{c} N-M\\ n-i \end{array}\right)}{\left(\begin{array}{c} N\\ n \end{array}\right)}$$

(2) $$\mathrm{Fold}\;\mathrm{change}\;=\frac{m}{M}\cdot \frac{N}{n}$$

where N is the total number of background genes, n is the total number of selected genes, M is the number of genes annotated to a specific GO term, and m is the number of selected genes annotated to that GO term. The *P*‐value was adjusted using the Benjamini‐Hochberg correction algorithm. GO enrichment analysis was performed on significant DEGs between control and infection groups (adjusted *P*‐value < 0.05) (Figures 2D, 5D and 6C, and Figure S3B,C, Supporting Information). Additionally, GSEA was conducted using the R package clusterProfiler (version 4.7.1.3).^[^ ^57^ ^]^ First, the FindMarkers function (parameters: only. pos = FALSE, logfc. threshold = 0, min. pct = 0) was used to obtain the *p*_val_adj values for all expressed genes. The DEGs were then ranked based on the ‐log10 (*p*_val_adj + 1e‐100), from largest to smallest. The GSEA function (parameters: minGSSize = 10, maxGSSize = 500, by = “fgsea”, nPermSimple = 10,000, pvalueCutoff = 1, pAdjustMethod = “BH”) was then applied to perform GSEA on the ranked gene list, with enriched terms having *P*.adjust < 0.05 considered significant. For Figure S6C (Supporting Information, ranked expressed genes between LPS + IFNγ and control il8^+^ neutrophils were subjected to GSEA.

### Scoring of Biological Processes

To evaluate the expression of gene sets representing specific GO pathways in individual cells (Figure 3D and Figure 6D,F), we employed Seurat's AddModuleScore function with default parameters to score each cell for target gene sets. Visualization was performed with Seurat's VlnPlot function. Statistical significance between groups was assessed using the Wilcoxon test.

### RNA Velocity‐Based Cell Fate Tracing

RNA velocity was used to infer the developmental trajectory, which represents the time derivative of spliced and unspliced mRNA in scRNA‐seq data. First, spliced and unspliced information was extracted from BAM files to construct a loom file using Velocyto (version 0.17.17).^[^ ^58^ ^]^ RNA splicing velocity and a velocity graph were then calculated using scVelo (version 0.2.3)^[^ ^59^ ^]^ and Dynamo (version 1.2.0). CellRank (version 2.0.1)^[^ ^31^ ^]^ was employed to compute initial and terminal states, as well as cell fate probabilities. Based on these results, we plotted directed lineages, identified putative lineage drivers, and visualized smooth gene expression trends (Figures 2E, 7C,F, and Figure S2C,D, Supporting Information).

### Pseudotime Analysis

Pseudotime trajectories were generated using Monocle (version 2.26.0)^[^ ^32^ ^]^ and Palantir (version 1.3.0)^[^ ^34^ ^]^ to infer potential lineage differentiation trajectories. For Monocle2 (Figures 3, 4, and 5E), the intersection of highly variable genes identified by Seurat and genes with high dispersion calculated by Monocle2 was used. Dimensionality reduction was performed using DDRTree, followed by cell ordering with Monocle2's default parameters. The ordered cells were visualized in a trajectory using the plot_cell_trajectory function, which plots the minimum spanning tree. Dynamic gene expression was visualized using the plot_genes_in_pseudotime function. A second pseudotime trajectory was inferred using Palantir (version 1.3.0) (Figure 2F; Figure S2A, Supporting Information), with the same root node (Neu‐rplp0) as in the Monocle analysis used to validate the robustness of the trajectories (Figure S2A, Supporting Information). Palantir was run with default parameters to construct the developmental trajectory of neutrophils. CytoTRACE (version 0.3.3)^[^ ^33^ ^]^ was used to infer the developmental trajectory of immune cell types. CytoTRACE is based on the concept that transcriptional diversity decreases during differentiation. The analysis was performed on a log2‐normalized expression matrix, and the predicted cell orders were projected onto the neutrophil UMAP space (Figure S2A, Supporting Information).

### Cell‐Cell Communication Analysis With CellPhoneDB

Cell‐cell communication analysis was performed using CellPhoneDB^[^ ^35^ ^]^ with normalized scRNA‐seq count matrices and cell‐type annotations. Significant ligand‐receptor interactions (*p* < 0.05) were identified through permutation testing (1,000 iterations) and visualized via heatmaps and network graphs, with validation using known interaction pairs and negative controls with shuffled labels.

### Regulon Network

The regulon network was explored using the Python package pySCENIC (version 0.12.1),^[^ ^40^ ^]^ which analyzed the co‐expression of TFs and their putative target genes. Due to the lack of a cisTarget database for Nile tilapia, a custom database was created. This involved obtaining nucleotide sequences of the 5′ UTR upstream and downstream 10 kb of protein‐coding genes from Ensembl, formatting a FASTA file, downloading the vertebrate motif collection from JASPAR,^[^ ^60^ ^]^ and converting it to Cluster‐Buster format. A custom cisTarget database was generated using the create_cistarget_motif_databases.py function. The raw count matrix was filtered to retain only genes present in the cisTarget database for use in the pySCENIC pipeline. The gene regulatory network was then constructed and scored using default parameters. The co‐expression network was built with the pyscenic grn function, potential regulons were identified with DNA motif analysis, and active gene networks were identified with the pyscenic ctx function. Regulon activity for each cell was calculated as the average normalized expression of the putative target genes (Figure 6E).

### Mellon Algorithm

For Figure 7D, we applied the Mellon algorithm^[^ ^61^ ^]^ to infer neutrophil‐state densities from high‐dimensional single‐cell data. First, diffusion maps were computed using Palantir. utils. run_diffusion_maps with PCA results (parameters: n_components = 30). We then used the Mellon. Density Estimator on the diffusion map eigenvectors (parameters: sc_subset. obs [“DM_EigenVectors”]) to predict log‐density values, which were assigned to the dataset (sc_subset. obs [“mellon_log_density”]) and clipped to the 5th and 100th percentile range (sc_subset. obs [“mellon_log_density_clipped”]) to mitigate outliers effects. This approach provided a robust characterization of cell‐state densities.

### Identification of Orthologous Gene Lists and Cross‐Species Mapping

To compare enriched gene lists across species and enable direct dataset mapping, reliable ortholog lists were essential. We began by identifying gene groups consisting of one‐to‐one, one‐to‐many, or many‐to‐many orthologs, as well as species‐specific genes, using the Ensembl multiple species comparison tool. For ortholog genes (one‐to‐many or many‐to‐many) within each group, we prioritized those with the highest homology confidence, using attributes in the following order: orthology. confidence, Gene. order. conservation. score, and Whole. genome. alignment. Coverage, accessed via biomaRt (https://grch37.ensembl.org/info/data/biomart/index.html). For genes that remained unmatched, we utilized DIAMOND^[^ ^62^ ^]^ to align Nile tilapia protein sequences with human protein sequences (Blast parameters: ‐f 6 –more‐sensitive –evalue 1e‐5). Higher‐confidence genes from the resulting alignments were selected based on the aforementioned criteria. Finally, we combined the DIAMOND alignment results with those obtained from Ensembl to ensure a comprehensive ortholog list. The IFNγ‐treated human neutrophil single‐cell RNA‐seq datasets were downloaded from NCBI under accession number PRJEB48995.^[^ ^28^ ^]^ For cross‐species analysis, we mapped the Nile tilapia data and the downloaded human data to our generated orthologous gene lists, creating matrices that included ortholog gene pairs expressed in both datasets (Figure 7H). We then used SCTransform to process the single‐cell data for both Nile tilapia and human. The SelectIntegrationFeatures function (parameter: nfeatures = 3000) was used to select features for integrating the multiple datasets. Finally, we used the Seurat CCA method for data integration, allowing us to unbiasedly identify similar cell types between the two species.

### Data Visualization

All plots were generated using R (version 4.2.0). In box plots, the boxes display the median (center line) and interquartile range (from the 25th to 75th percentile), with whiskers representing 1.5 times the interquartile range and circles indicating outliers. In violin plots, the gray line on each side represents a kernel density estimation, illustrating data distribution; wider sections indicate higher probability, while thinner sections indicate lower probability.

### Statistical Analysis

R (version 4.2.3) was used for scRNA‐seq data analysis, and GraphPad Prism (version 10.0) was used for graphical processing. The in vivo experiments were independently repeated at least three times. Statistical analyses between two groups were performed using an unpaired two‐tailed Student's* t*‐test, and data is mean ± SEM in GraphPad Prism 10.0.

## Conflict of Interest

The authors declare no conflict of interest.

## Author Contributions

X.Z., M.Z., K.L., and W.L. contributed equally to this work. X.Z. and P.H. conceived the study. X.Z. conducted most of the experiments. M.Z. and P.H. performed computational analysis. K.L. performed the *S. agalactiae* infection and flow cytometry experiments. M.Z. and W.L. assisted with scRNA‐Seq library preparation. W.L. and S.H. maintained and prepared the tilapia materials. X.L. developed the interactive web portal. K.L., W.G., J.Z., and J.Y. contributed to the recombinant protein preparation. M.L., Z.W., and L.C. contributed reagents and aided in the experimental design. X.Z., M. Z., and P.H. analyzed the results and wrote the manuscript with inputs from all authors. P.H. supervised the study.

## Supporting information



Supporting Information

## Data Availability

The data that support the findings of this study are available from the corresponding author upon reasonable request.
