# Supplementary material for: Single‐Cell Transcriptome Profiling Reveals Conserved IFNγ‐IL8 Signaling‐Induced Antibacterial Neutrophil States during Bacterial Infection
Source: Adv Sci (Weinh). 2025 Jul 1;12(37):e04840. doi: 10.1002/advs.202504840 (PMC12499466; doi:10.1002/advs.202504840)
Supplement: Supplementary file 1 — Supporting Information [file ADVS-12-e04840-s001.docx]

**Supplementary Materials**


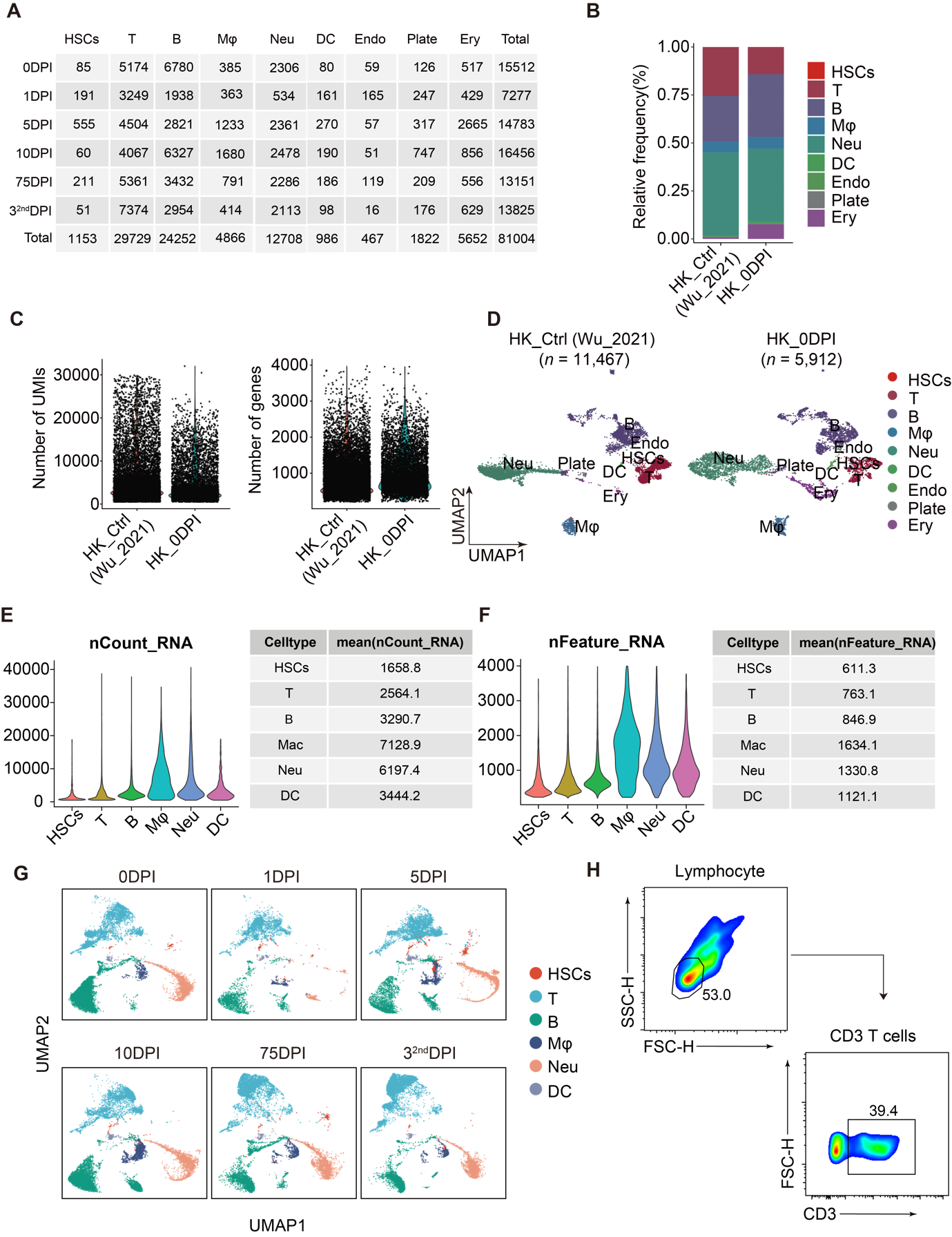


**fig. S1. Quality control analysis.**

(**A**) Summary table of cell numbers after quality control. HSCs: hematopoietic stem cells, T: T cells, B: B cells, Mφ: macrophages, Neu: neutrophils, DC: dendritic cells, Endo: endothelial cells, Plate: platelet, Ery: erythrocyte. DPI: days post-infection. (**B**) Bar plot showing immune cell compositions of HK immune cells in our data and published data (Wu_2021)[16]. Cell subtypes are color-coded. HK_ctrl: head kidney_control. (**C**) Violin plots of the number of genes and number of UMIs in our control immune cells from the head kidney compared with control immune cells from the head kidney of Wu_2021 data [16]. HK: head kidney; DPI: days post-infection. (**D**) UMAPs of HK immune cells in our data and published data (Wu_2021) [16], colored by dataset or cluster identity. Cluster labels were transferred from our data to published data (Methods). (**E**)Violin plot and table showing the average unique molecular identifiers (nUMI) per cell type and (**F**) showing the average number of genes (nGene) per cell type. (**G**) UMAP plots showing the composition and similarities of major cell types at each time point. Color represents the cell type. DPI: days post-infection. (**H**) The strategy of gating for flow cytometry data (related to Fig.1I).


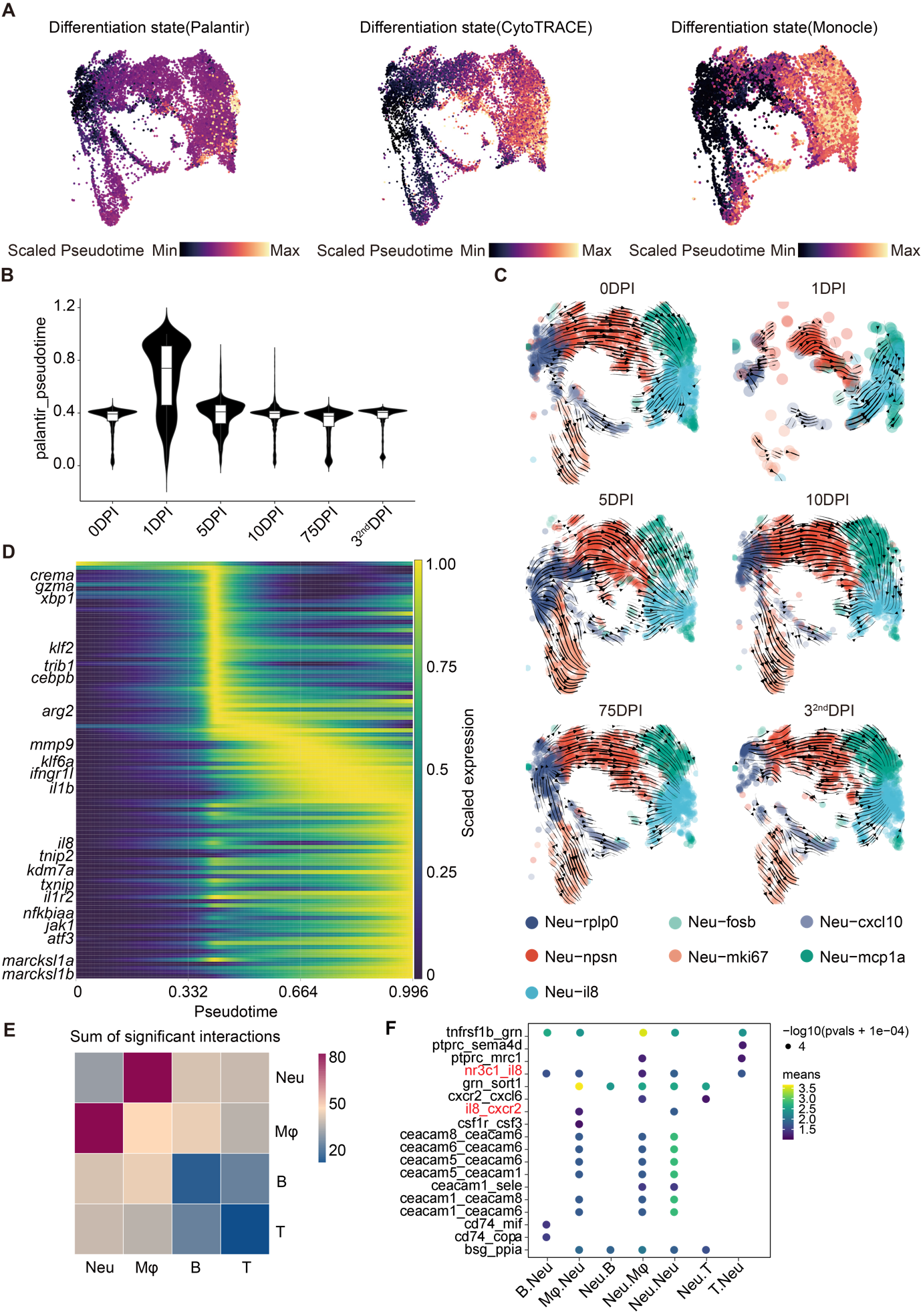


**fig. S2. Neu-il8 subset is terminally differentiated.**

(**A**) Differentiation state of neutrophil subsets estimated by Palantir, CytoTRACE, and Monocle. Colors indicate scaled pseudotime. (**B**) Violin plot illustrating the pseudotime distribution at each time point inferred using the Palantir algorithm in CellRank. (**C**) UMAP visualization of neutrophil trajectories using RNA velocity analysis, split by time points. The streamlines indicate the integration paths connecting local projections from the observed state to the extrapolated future state. Arrows indicate the directionality of subtype transitions. Cell subtypes are color-coded. (**D**) Heatmap of DEGs, ordered based on their common kinetics through pseudotime using CellRank. Immune-related genes were marked. (**E**) Heatmap show the number of significant interactions among four immune cell types at 1 DPI following *S. agalactiae* infection in tilapia predicted by CellphoneDB. (**F**) Dot plot shows the expression of receptor-ligand gene pairs with significant interactions in neutrophils and other cell groups at the same time point. Dot size indicates the significance of the receptor-ligand interaction, while color represents the average expression level of the gene pair in the interacting cell populations. T: T cells, B: B cells, M: macrophages, Neu: neutrophils.


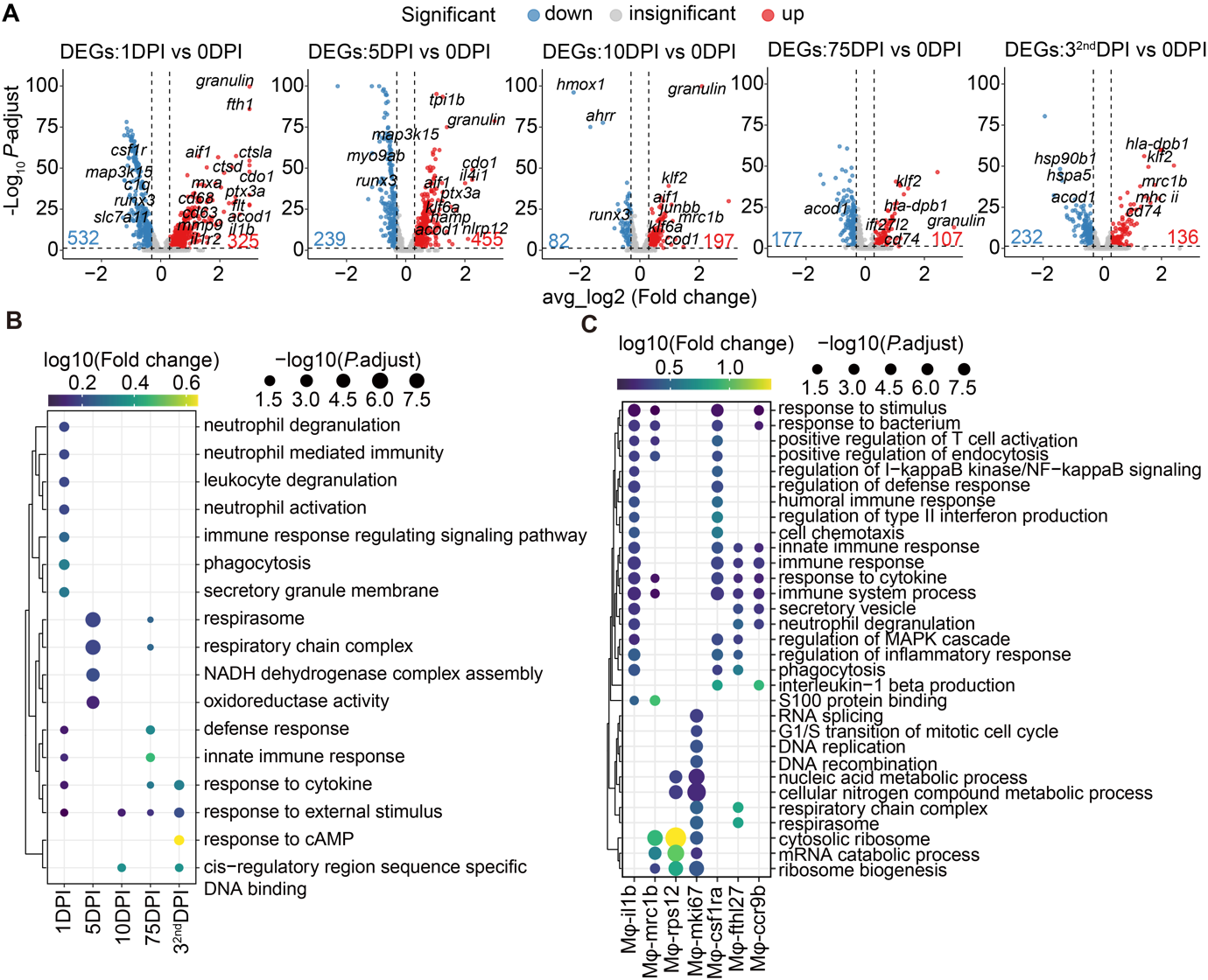


**fig. S3. Function and differentiation analysis of macrophage subsets.**

(**A**) Volcano plots showing genes differentially expressed in macrophages between five time points and the control group (0 DPI). Genes significantly upregulated (red) or downregulated (blue) at each time point were identified by a two-sided Wilcoxon rank-sum test (Bonferroni-adjusted *P*-value < 0.05), with selected immune-related genes labeled. DEGs: differentially expressed gene. DPI: days post-infection. (**B**) Dot plot showing GO enrichment of marker genes for each time point. The significance of enrichment was determined by a hypergeometric test. Dot size is scaled by −log10 (FDR-adjusted *P*-value) and colored by log10 (fold change). Fold change is calculated as the fraction of DEGs found in a pathway divided by the fraction of all expressed genes within the pathway. (**C**) Dot plot showing GO enrichment of marker genes for each macrophage subtype. The significance of enrichment was determined by a hypergeometric test. Dot size is scaled by −log10 (false discovery rate (FDR)-adjusted *P*-value) and colored by log10 (fold change). Fold change is calculated as the fraction of DEGs found in a pathway divided by the fraction of all expressed genes within the pathway.


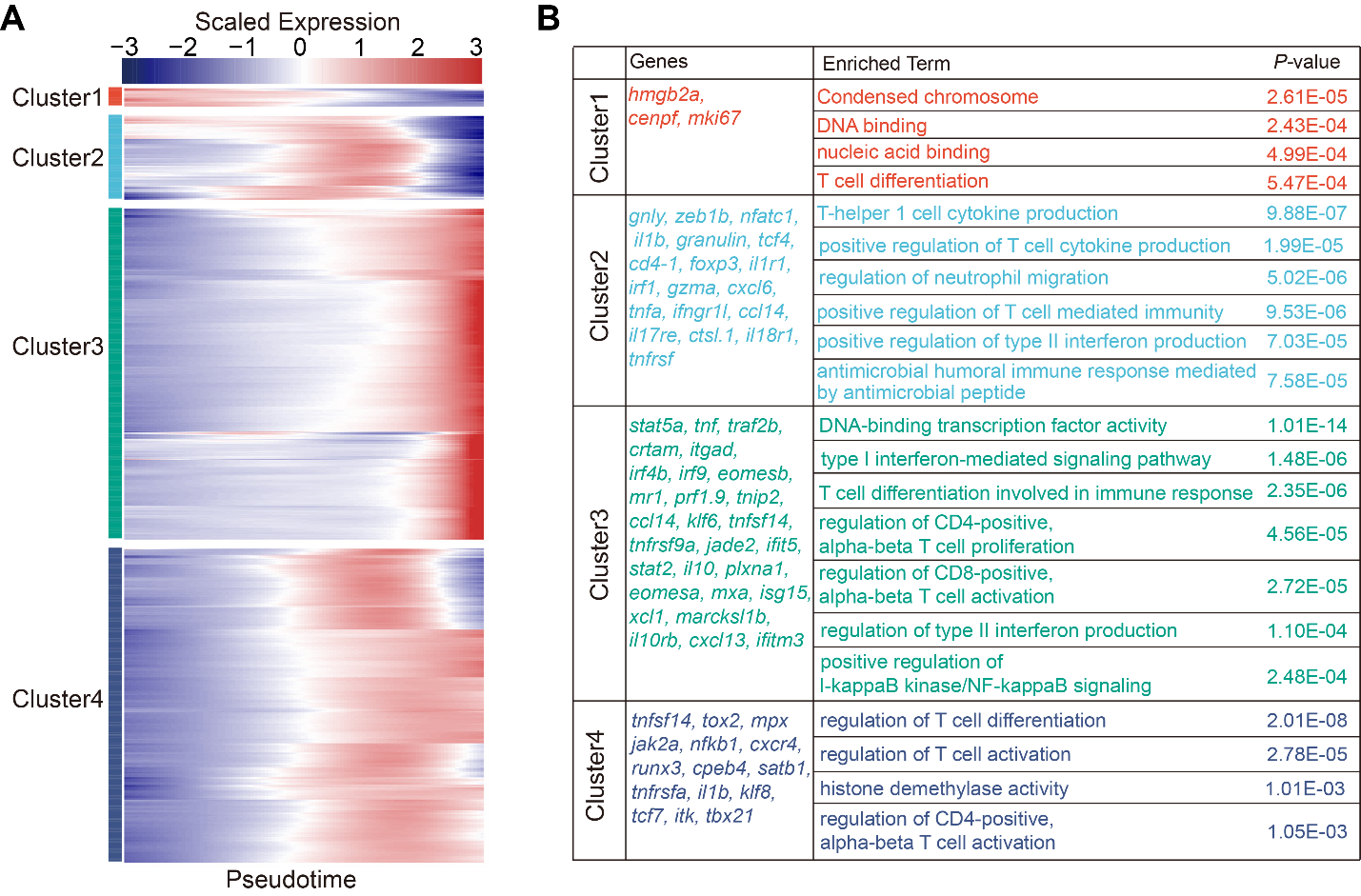


**fig. S4. Differentiation analysis of T cell subsets.**

(**A**) Heatmap of DEGs of T cell states, ordered based on their common kinetics through pseudotime using Monocle. Clusters were identified by hierarchical clustering. Colors indicated the expression of DEGs. (**B**) DEGs, enriched GO terms, and their *P*-values associated with each model are shown. The significance of enrichment was determined by a hypergeometric test.


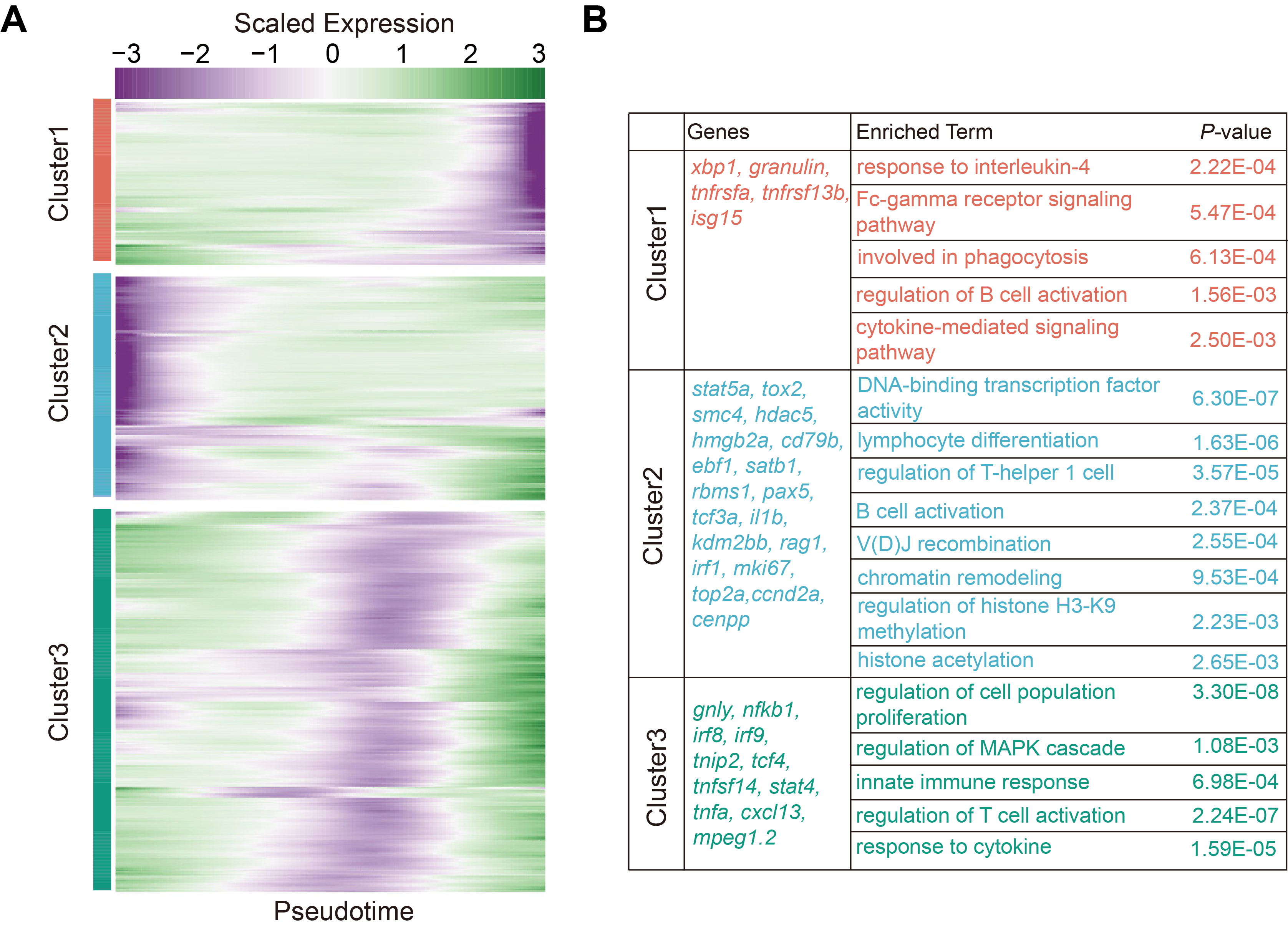


**fig. S5. Differentiation analysis of B cell subsets.**

(**A**) Heatmap of DEGs of B cell states, ordered based on their common kinetics through pseudotime using Monocle. Clusters were identified by hierarchical clustering. Colors indicated the expression of DEGs. (**B**) DEGs, enriched GO terms, and their *P*-values associated with each model are shown. The significance of enrichment was determined by a hypergeometric test.


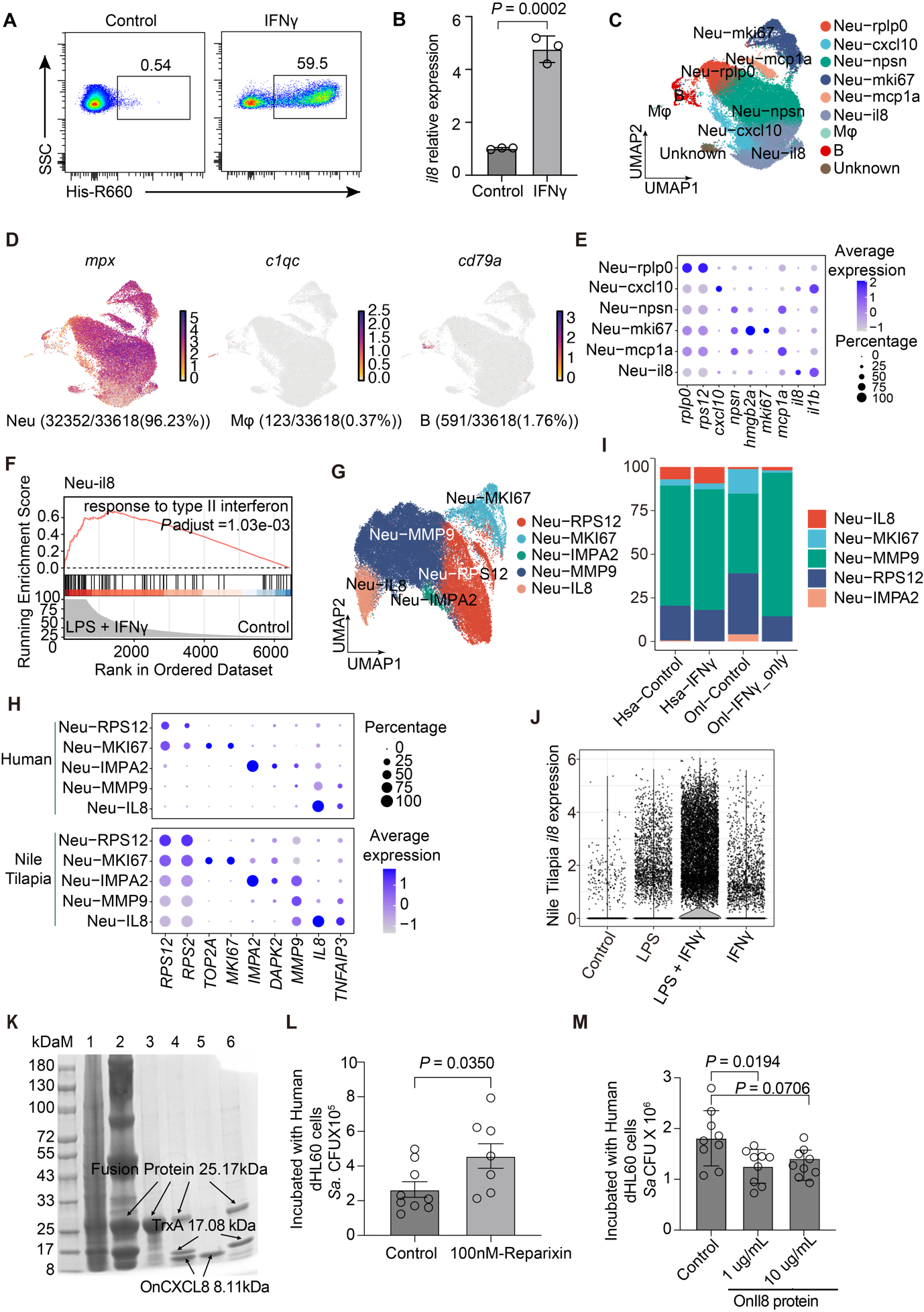


**fig. S6. Identification subset analysis of control, LPS- and IFNγ-stimulated neutrophils.**

(**A**) His-tagged recombinant IFNγ stimulated tilapia neutrophils and verified surface binding via FACS using anti-His antibodies. (**B**) qPCR analysis showed that IFNγ stimulation significantly upregulated IL8 expression in neutrophils. Data are mean ± SEM, each dot represents an individual animal (*n* = 3). Significance was determined by an unpaired two-tailed Student’s *t*-test. (**C**) UMAP visualization of integrated 7,015 control neutrophils, 11,814 LPS-stimulated neutrophils, and 13,523 IFNγ-stimulated neutrophils. Cell types are color-coded. Mφ: macrophages, B: B cells. (**D**) UMAP visualization of classical marker genes of neutrophils (*mpx*), macrophages(*c1qc*), and B cells (*cd79a*), with cells colored according to the normalized expression level of the corresponding genesa. Neu: neutrophils, Mφ: macrophages, B: B cells. (**E**) Dot plot showing the expression pattern of top marker genes for neutrophil subtypes (related to A). Colors represent the average expression of each gene in each cluster, scaled across all clusters. Dot size indicates the percentage of cells in each cluster expressing the corresponding gene. (**F**) GSEA of “response to type II interferon” pathway in IFNγ-stimulated neutrophils compared to the control group of Neu-il8 subtype. Significance was determined using a permutation test, and the *P*-value was adjusted with the Benjamini-Hochberg Correction. Genes were ranked according to their significance. **(G)** UMAP visualization of integrated tilapia and human scRNA-seq datasets. The raw human datasets, including control and IFNγ-stimulated neutrophil samples, were sourced from Montaldo et al [28]. Cell types are color-coded. (**H**) Dot plot showing the expression pattern of top marker genes for neutrophil subtypes from tilapia and human scRNA-seq datasets [28] from control and IFNγ-treated CD16^+^ neutrophils from cord blood samples (related to D). Colors represent the average expression of each gene in each cluster, scaled across all clusters. Dot size indicates the percentage of cells in each cluster expressing the corresponding gene. (**I**) Bar plot showing the proportions of neutrophil subtypes across unstimulated and IFNγ-stimulated conditions in humans and tilapia. Cell types are color-coded. (**J**) Violin plots showing the distribution of *il8* gene expression levels under different treatment conditions. The boxplots inside each violin represent the interquartile range, with the midline indicating the median. Dots represent individual cell. (**K**) The prokaryotic recombinant expression and purification of Il8 in Nile Tilapia. M: Protein Marker (8-180 kDa), 1: Supernatant of expression culture, 2: Protein eluted with 500 mM imidazole, 3: Protein purified by gel filtration chromatography + anion exchange chromatography, 4: Protein after enterokinase digestion, 5: Flow-through protein from anion exchange chromatography - target protein, 6: Protein eluted from anion exchange chromatography. (**L**) Scatter plot showing the *S. agalactiae* CFU recovered from control and CXCR1/2 inhibitor reparixin (IL8 pathway blocke)-stimulated dHL-60 cells. (**M**) Scatter plot showing the *S. agalactiae* CFU recovered from control and OnIl8-stimulated dHL-60 cells. *S. agalactiae* was incubated with control and Il8 -stimulated immune cells for 5 h, and then plated on BHI agar for CFUs enumeration.


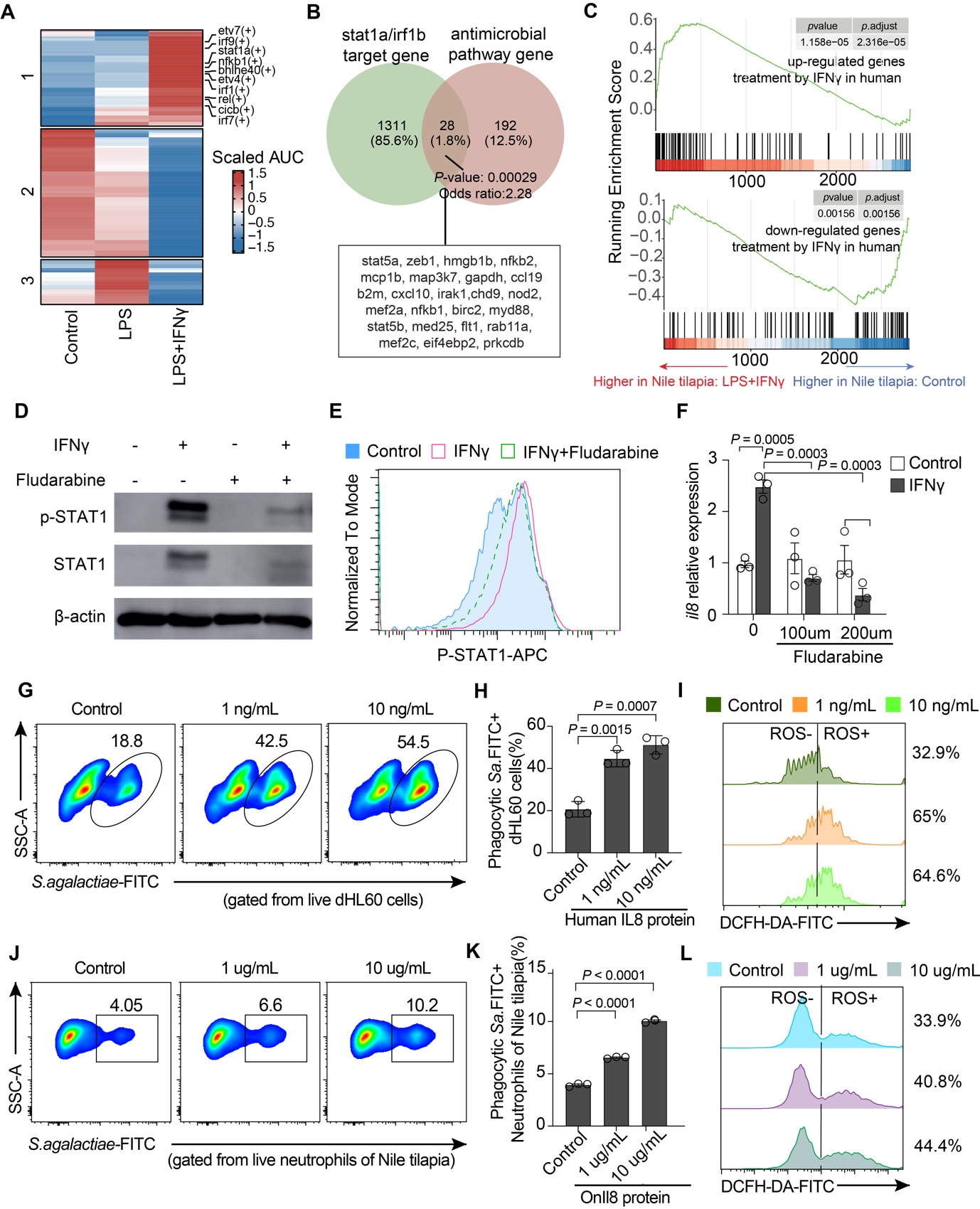


# fig. S7. Antibacterial mechanisms of IFNγ-IL8 axis.

(**A**) Heatmap showing the scaled regulon activities of transcription factor genes under each treatment condition. AUC, area under the curve. (**B**) Venn diagram shows the overlap between downstream target genes of *stat1a* and *irf1b* inferred by SCENIC and genes involved in antimicrobial pathways. *P*-value < 0.05 indicates that the downstream target genes of *stat1a/irf1b* are significantly enriched in antimicrobial pathways. (**C**) Gene set enrichment analysis revealed that genes upregulated in human neutrophils upon IFNγ stimulation were significantly enriched among the genes upregulated in tilapia neutrophils following LPS + IFNγ treatment (top panel). Similarly, genes downregulated in human neutrophils after IFNγ stimulation were significantly enriched among the genes downregulated in tilapia neutrophils treated with LPS + IFNγ (bottom panel). The x-axis shows the differentially expressed genes ranked by fold change (with genes upregulated in the LPS + IFNγ group on the left, and those upregulated in the control group on the right). The green curve depicts the enrichment score profile, and the black vertical lines indicate the positions of gene set members within the ranked list. The *P*-value was adjusted with the Benjamini-Hochberg Correction. (**D**) Western blotting showing the protein level of STAT1 and P-STAT1 in dHL-60 with Fludarabine and IFNγ treatment. (**E**) p-STAT1 level was assessed by flow cytometry in dHL-60 with Fludarabine and IFNγ treatment. (**F**) qPCR analysis showing the il8 expression level in dHL-60 with IFNγ and different concentrations of Fludarabine treatment. Data in bar plots are mean ± SEM, each dot represents an individual animal (*n* = 3). Significance was determined by an unpaired two-tailed Student’s *t*-test. (**G-H**) Representative flow cytometry plots (**G**) and bar plots (**H**) showing the frequency of phagocytic dHL-60 cells stimulated with IL8 protein. Data in bar plots are mean ± SEM, each dot represents an individual animal (*n* = 3). Significance was determined by an unpaired two-tailed Student’s *t*-test. (**I**) Flow cytometry analysis of intracellular ROS levels using DCFH-DA in the dHL-60 cells. Representative histograms showing DCF fluorescence in untreated (dark green), IL8 (1 ng/mL)-treated (yellow), and IL8 (10 ng/mL)-treated (light green) cells. Quantification of mean fluorescence intensity (MFI) using FlowJo software. (**J-K**) Representative flow cytometry plots (**J**) and bar plots (**K**) showing the frequency of phagocytic tilapia neutrophils stimulated with Il8 protein. Data in bar plots are mean ± SEM, each dot represents an individual animal (*n* = 3). Significance was determined by an unpaired two-tailed Student’s *t*-test. (**L**) Flow cytometry analysis of intracellular ROS levels using DCFH-DA in the tilapia neutrophils. Representative histograms showing DCF fluorescence in untreated (blue), Il8 (1 μg/mL)-treated (purple), and Il8 (10 μg/mL)-treated (dark green) cells. Quantification of mean fluorescence intensity (MFI) using FlowJo software.
